# Randomised controlled trial of fluoxetine versus naltrexone in compulsive sexual behaviour disorder

**DOI:** 10.1136/bmjment-2026-302953

**Published:** 2026-10-06

**Authors:** Josephine Savard, Marta Piwowar, Roberth Adebahr, Markus Byström, Jonas Hallberg, Cecilia Dhejne, Katarina Görts Öberg, Jussi Jokinen

**Affiliations:** 1 Department of Medicine, Huddinge, Karolinska Institute, Stockholm, Sweden; 2 Department of Clinical Sciences (Psychiatry), Umeå University, Umeå, Sweden; 3 ANOVA, Karolinska University Hospital, Stockholm, Stockholm County, Sweden; 4 Centre for Psychiatry Research, Department of Clinical Neuroscience, Karolinska Institute, Stockholm, Sweden

**Keywords:** Psychiatry, Psychopharmacology

## Abstract

**Background:**

Compulsive sexual behaviour disorder (CSBD), characterised by preoccupation with and loss of control over sexual behaviours, is associated with negative consequences in several areas of life. Evidence for pharmacological treatment remains limited, with only two previous randomised controlled trials and no studies comparing fluoxetine and naltrexone.

**Objective:**

To investigate whether naltrexone was more efficient and tolerable than fluoxetine in the treatment of CSBD.

**Methods:**

In this open-label superiority trial, 80 participants diagnosed with CSBD according to International Classification of Diseases-11 criteria were randomly assigned (1:1) to receive fluoxetine (starting dose 20 mg/day) or naltrexone (starting dose 25 mg/day) for 8 weeks followed by 6 weeks without treatment. Symptom severity was recurringly assessed using the Hypersexual Disorder: Current Assessment Scale (HD:CAS). Linear mixed models were used for intention-to-treat and per-protocol analyses of treatment effects.

**Findings:**

79 men and 1 woman were randomised to receive fluoxetine (n=40) or naltrexone (n=40). Analysis showed no statistically significant difference in HD:CAS score reduction between treatments at primary endpoint; naltrexone was not superior to fluoxetine. However, participants in the two treatment groups showed different patterns of symptom improvement over time.

Two participants (5.1%) receiving fluoxetine were withdrawn due to adverse events (urticaria and elevated liver enzymes) and four participants (10.3%) discontinued treatment; six participants (15%) receiving naltrexone discontinued treatment.

**Conclusions:**

Although no statistically significant between-group difference was observed at week 8, symptom trajectories and adverse effect profiles differed between groups.

**Clinical implications:**

Symptom-change patterns and adverse effect profiles may guide individualised treatment decisions.

**WHAT IS ALREADY KNOWN ON THIS TOPIC**

- Compulsive sexual behaviour disorder (CSBD) is a recently introduced diagnosis in the WHO’s classification system, estimated to affect approximately 2–3% of the population.
- CSBD is characterised by a persistent inability to control sexual impulses or urges, resulting in significant distress or negative consequences in various areas of life.
- Evidence for pharmacological treatment of CSBD is limited, with only two previous randomised controlled trials and no studies comparing fluoxetine and naltrexone.

**WHAT THIS STUDY ADDS**

- This is the first randomised controlled trial directly comparing fluoxetine and naltrexone for the treatment of CSBD.
- No significant difference in overall symptom reduction was observed between naltrexone and fluoxetine after 8 weeks of treatment; however, the course of symptom improvement varied over time across the study groups.
- Naltrexone and fluoxetine demonstrated different adverse effect profiles, suggesting that treatment decisions may benefit from individualised assessment of tolerability and patient preference.

**HOW THIS STUDY MIGHT AFFECT RESEARCH, PRACTICE OR POLICY**

- Future studies should further examine the course of symptom change observed within each treatment group and establish validated measures for assessing treatment-related change in CSBD.
- Treatment decisions may be guided by patterns of symptom change, adverse-effect profiles, alongside patient characteristics and preferences, rather than expectations of superior efficacy.
- Future research should focus on long-term outcomes, treatment continuation strategies and identify patient subgroups that may respond differently to these interventions.

## Background

 Compulsive sexual behaviour disorder (CSBD), as defined in the 11th edition of the International Classification of Diseases (ICD-11), is characterised by a persistent pattern of failure to control sexual impulses and behaviours often associated with adverse consequences (eg, negative impacts on health or relationships, and elevated risk of sexual offending), resulting in marked distress and/or significant impairment in important areas of functioning.^1 2^ Nationally representative German data suggest a 12-month prevalence of 3.2% in men and 1.8% in women,^3^ highlighting the need for accessible and evidence-based treatment approaches.

The World Federation of Societies of Biological Psychiatry (WFSBP) guidelines for CSBD recommend psychoeducation and psychotherapy as the first line of treatment.^4^ If insufficient results, selective serotonin reuptake inhibitor (SSRI, eg, fluoxetine 20–80 mg/day) or the opioid antagonist naltrexone (50–200 mg/day) may be added. SSRIs may be suitable for CSBD patients with obsessive-compulsive patterns, such as intrusive sexual thoughts and relief through sexual behaviours as suggested by their established efficacy in treating mood and anxiety disorders. Naltrexone may be especially appropriate for individuals with CSBD and co-occurring substance use disorders and addictive behavioural patterns.^4 5^ The drug is proposed to potentially reduce sexual cravings and the drive to seek sex-related reinforcement by affecting the brain’s reward system.^6^ Despite the drugs potentially targeting different CSBD profiles, patients often present with overlapping or insufficiently distinct clinical features, making it difficult to assign them clearly to one category in routine clinical practice.

Adverse effects associated with SSRIs are usually mild and transient,^7^ though sexual dysfunctions are less likely to reduce over time.^8^ Such adverse sexual effects may be used as a mechanism of treatment, however the inclusion of anticipated sexual side effects in the treatment rationale is considered controversial.^5^ In our 8-week pilot study of naltrexone in 20 men with CSBD, participants reported CSBD symptom improvement, with mild to moderate adverse effects that mostly subsided after the first week.^9^

While the WFSBP guidelines can serve as a valuable resource, it is important to acknowledge that there is still scarce evidence for pharmacotherapy, with only two randomised controlled trials (RCTs),^10 11^ and most other studies being case-reports or case-series with diagnostic or intervention confounders.^5^ Given the impact and prevalence of CSBD, it is of utmost importance to provide effective treatments.

## Objective

Our primary aim was to test whether naltrexone (defined as alternative treatment) was more effective than the SSRI fluoxetine (defined as standard treatment) in reducing symptoms of CSBD, as no previous study has directly compared these drugs. We also aimed to compare adverse events (AEs), treatment adherence, number of dropouts and participants’ wish to resume treatment.

## Methods

### Study design

This 14-week open-label, randomised controlled parallel-group superiority trial was conducted at ANOV, an outpatient centre for sexual medicine, andrology and trans medicine at Karolinska University Hospital, Stockholm, Sweden. The trial was pre-registered (EudraCT no. 2019–0 04 255-36) and accessible at https://www.clinicaltrialsregister.eu.

The trial procedures were carried out in accordance with the Declaration of Helsinki and monitored by an independent trial unit. One minor protocol change was implemented after trial commencement regarding an update on data management according to General Data Protection Regulation (17 December 2021). The participants and public were not involved in the design of the trial. The trial was academically initiated, financed by grants and entirely independent of the pharmaceutical manufacturers. The data supporting the findings of this trial are available from the corresponding author on reasonable request.

### Participants

The trial included individuals meeting the ICD-11 criteria for CSBD, aged 18–66 years. Individuals with severe physical illness, mental health condition or substance use disorders were excluded. Individuals likely to meet eligibility criteria (online supplemental table S1) received information about the trial through PrevenTell, a Swedish national helpline for problematic sexuality.^12^ Given the substantial comorbidity between CSBD and paraphilic interests and disorders reported in previous research,^12 13^ individuals with paraphilic interests or disorders were eligible for inclusion, provided that CSBD was their primary presenting problem and reason for seeking treatment, reflecting the clinical population encountered in routine practice. Potential participants completed online questionnaires and were subsequently invited to the clinic for assessment.

### Randomisation and masking

The allocation sequence, which was concealed to the enrolling personnel, was generated in blocks of 4 and 6 by an independent clinical trial support unit, The Karolinska Trial Alliance, using a web-based tool. A research nurse randomised participants to receive either fluoxetine (n=40) or naltrexone (n=40) using opaque, sealed envelopes.

### Procedures

Screening visits included collecting a written informed consent, blood and urine sampling and two consecutive assessments; a board-certified psychiatrist assessed medical, sexual and psychiatric history as well as conducted the Mini International Neuropsychiatric Interview 7.0.0^14^ and a psychologist conducted a structured interview focusing on sexual behaviours. Eligibility was independently assessed, discrepancies led to exclusion. No compensation was offered. For a detailed account of screening procedures, assessments and the complete eligibility criteria, see eMethods in online supplemental file and the published study protocol.^15^

The primary endpoint was at week 8, followed by 6 weeks without treatment before the end of the trial at week 14. Participants allocated to fluoxetine started with an initial dose of 20 mg/day which was augmented to 40 mg/day after 4 weeks if the medication was tolerated without satisfactory symptom reduction. At week 7 these participants reverted to 20 mg/day for a washout period prior to the end of treatment. The initial dose for those assigned naltrexone was 25 mg/day, which was increased to 50 mg/day after 3–5 days if tolerated. Symptoms were assessed at regular intervals using online self-report questionnaires and monitored by the trial psychiatrist (online supplemental figure 1). Blood samples were collected for safety reasons at week 4 and at end of treatment. Pre-existing prescribed drugs were permitted if maintained at a stable dose. Initiation of non-compatible drugs, pregnancy or initiation of psychotherapy during the treatment period led to discontinuation from the trial.

### Outcomes

#### Primary outcome

The Hypersexual Disorder: Current Assessment Scale (HD:CAS)^16^ measures symptom severity during the preceding 2 weeks according to the conceptualisation of hypersexual disorder suggested for the Diagnostic and Statistical Manual of Mental Disorders, Fifth Edition (DSM-5).^17^ The scale assesses CSBD-related behaviours, perceived control, risk and harm. Six items are scored on a Likert scale (from 0 to 4), with a total score of 0–24. HD:CAS has been used for both pharmacological and therapeutic studies by our research team and has been deemed sensitive to detect symptom changes.^9 18 19^ The HD:CAS is provided in the online supplemental file.

Since there are no validated symptom change scales for CSBD, additional CSBD measures were used. A full description of measures can be found in the trial protocol and in the eMethods in online supplemental file.

#### Secondary outcomes

Tolerability was evaluated non-systematically throughout all time points and systematically at week 8 with the Udvalg for Kliniske Undersøgelser side effect rating scale, which measures 48 symptoms.^20^ Harms deemed as possibly or probably related to the intervention were coded using the Medical Dictionary for Regulatory Activities system, V.27.1. The severity of treatment-emergent AEs was assessed using the US National Cancer Institute Common Terminology Criteria for Adverse Events V.5.0.

At the end of treatment, blood samples were collected from fluoxetine participants to assess concentrations of fluoxetine and norfluoxetine. Due to the COVID-19 pandemic, the Karolinska research laboratory was unable to prioritise the corresponding analysis of naltrexone concentration. Participants were encouraged to return any unused tablets for a medication count. Other secondary outcomes included number of dropouts and participants’ wish to resume pharmacological treatment at the end of the trial (with the options: yes/no/do not know).

### Statistical analysis

The power calculation, based on HD:CAS data from our previous study,^18^ indicated that a sample size of 68 would be required to detect a clinically significant difference with a power of 80% and a significance level of 0.05. To account for dropouts, 80 participants were set to be randomised.^15^

Descriptive statistics summarised baseline characteristics (frequencies and percentages). Normality was assessed using the Shapiro-Wilk test; non-normally distributed variables were reported with median and IQR and normally distributed data with mean, SD and range.

Both intention-to-treat (ITT) and per-protocol (PP) analyses were conducted. The ITT analyses included all randomised participants (n=40 per group). The PP analyses only included those who completed treatment and assessments at week 8 (n=66, 32 in the fluoxetine group and 34 in the naltrexone group). Linear mixed models (LMM) were used for the ITT and PP analyses of the treatment effects. The model was expressed as follows: yij=β0+β1(Time)+β2(Treatment)+β3(Time×Treatment)+bi+ℇij.

Time was defined as a categorical variable, with each time point representing a measurement occasion. The baseline measurements served as the reference; to account for individual differences, each participant’s baseline measurements were considered as random intercepts. The interaction variable was used to analyse changes over time across treatment. The same analyses were conducted as sensitivity analyses using two additional CSBD questionnaires. Post hoc tests to compare means used the Tukey method. No imputation was conducted, as LMM are not sensitive to missing data given the assumption that data are missing at random.

χ^2^ was calculated to compare the number of dropouts and wishes to continue treatment between treatments. In exploratory sensitivity analyses addressing baseline imbalances in paraphilic disorders and history of substance use disorders, Mann-Whitney U tests were used to compare continuous baseline measures. All statistical analyses were performed using R V.4.4.1 and SPSS V.29.0 (IBM, Armonk, New York, USA). The statistician who performed the data analysis was blinded to the group allocation. No interim analyses were conducted.

## Findings

### Enrolment

The trial included the first participant on 02 November 2020, and the last participant’s final visit was on 18 June 2024. Of 172 individuals providing online informed consent, 102 were screened on site and 80 were randomised (figure 1). Baseline characteristics of those withdrawing before the on-site assessment are detailed in online supplemental table 2. The most common exclusion reason at the on-site assessment was not meeting criteria for CSBD. The median HD:CAS score at baseline for the excluded group (n=22) was 10.5 points (IQR 7.75–14.25). 1 participant randomised to fluoxetine was excluded before initiating treatment due to elevated blood glucose levels, resulting in a final cohort of 79 participants.

**Figure 1:**
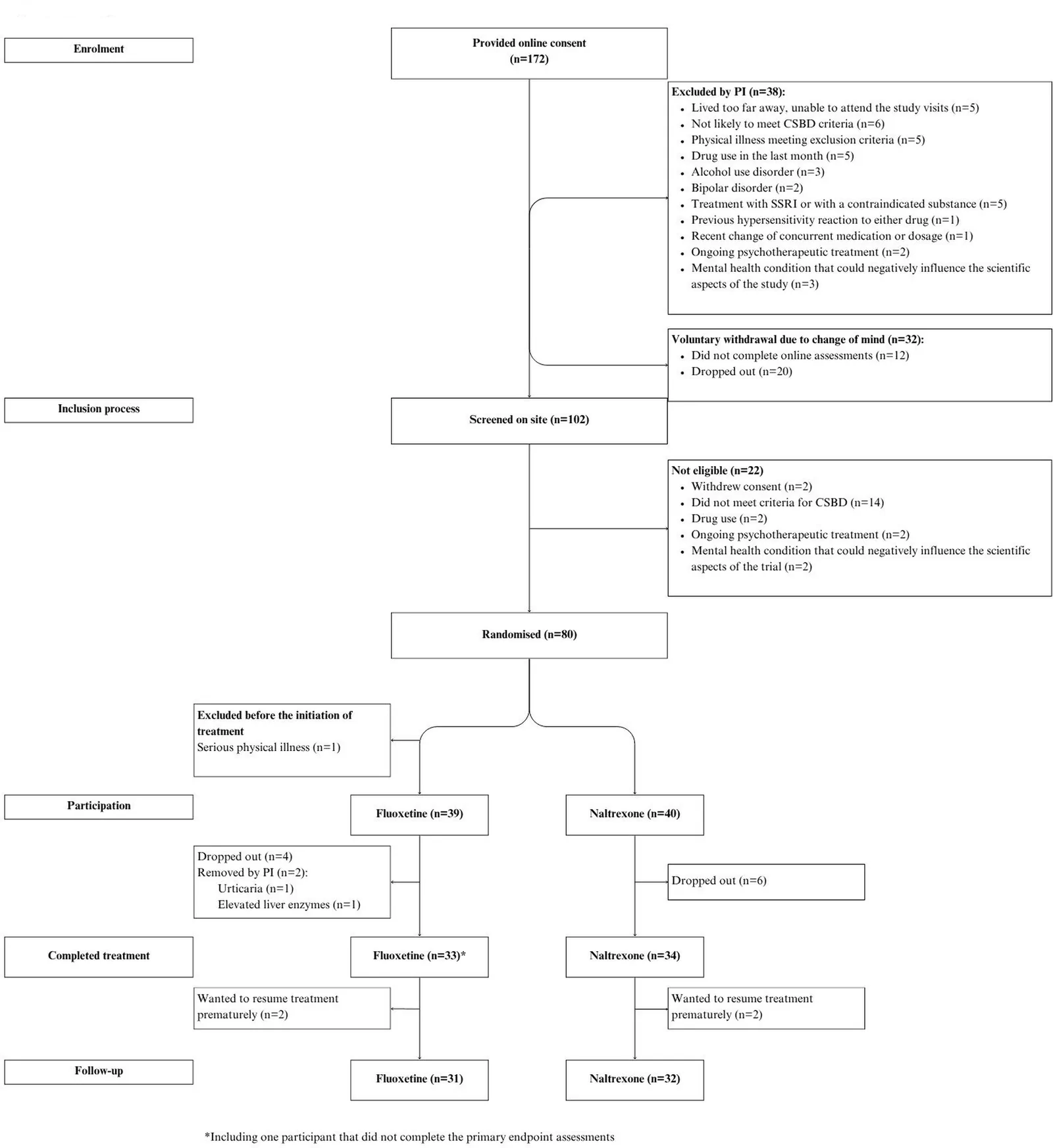
Flow chart. CSBD, compulsive sexual behaviour disorder; PI, principal investigator; SSRI, selective serotonin reuptake inhibitor.

### Baseline characteristics

Participants’ mean age was 36.51 years (SD=10.94) and all but one were men (79/80, 99%, table 1). The majority of participants were in a steady relationship (61/80, 76%), and identified as exclusively heterosexual (46/80, 58%). The median HD:CAS score at baseline was 14 points (IQR 12–16), and participants had on average experienced CSBD-symptoms for 15.74 years (SD=9.76). Most reported both solitary and non-solitary problematic sexual behaviours (51/80, 64%). Paraphilic interests were reported by 40% of participants (32/80), with 13% (10/80) meeting the criteria for at least one paraphilic disorder. 64% (51/80) were assessed as having a previous or current psychiatric condition, with depression and previous substance use disorder being the most frequent.

**Table 1:** Baseline participant characteristics (N=80)

Characteristic	Fluoxetine (n=40)*	Naltrexone (n=40)*
Age in years, mean (SD)	37.40 (10.91)	35.63 (11.04)
Range	19−63	21−63
Gender, No. (%)		
Man	40 (100)	39 (98)
Woman	0 (0)	1 (3)
Other	0 (0)	0 (0)
Place of birth, No. (%)		
Sweden	34 (85)	33 (83)
Other country	6 (15)	7 (18)
Biological parent(s) born abroad, No. (%)		
Yes	12 (30)	12 (30)
No	28 (70)	28 (70)
Ever had steady relationship (at least 1 month), No. (%)		
Yes	39 (98)	39 (98)
No	1 (3)	1 (3)
Steady relationship past month, No. (%)		
Yes	29 (73)	32 (80)
No	10 (25)	7 (18)
Living situation, last month, No. (%)		
Living alone	9 (23)	11 (28)
Living with spouse/partner(s)	26 (65)	26 (65)
Living with parents	4 (10)	3 (8)
Living with other adults	1 (3)	0 (0)
Children living at home, No. (%)		
Yes	22 (55)	19 (48)
No	18 (45)	21 (53)
Occupation, No. (%)		
Employed/working	33 (83)	32 (80)
Unemployed	2 (5)	2 (5)
Studying	4 (10)	6 (15)
Other	1 (3)	0 (0)
Sexual orientation, No. (%)†		
Heterosexual	23 (58)	23 (58)
1	12 (30)	11 (28)
2	4 (10)	2 (5)
3	0 (0)	2 (5)
4	0 (0)	1 (3)
5	0 (0)	1 (3)
Homosexual	1 (3)	0 (0)
Other	0 (0)	0 (0)
M.I.N.I., No. (%)		
Depression		
Current	4 (10)	3 (8)
Past	13 (33)	12 (30)
Recurrent	4 (10)	2 (5)
Suicide risk		
Low	37 (93)	36 (90)
Moderate	1 (3)	3 (8)
High	2 (5)	1 (3)
Manic episode	0 (0)	0 (0)
Panic disorder		
Current	1 (3)	1 (3)
Previous	1 (3)	2 (5)
Social anxiety disorder	3 (8)	2 (5)
Obsessive compulsive disorder	0 (0)	0 (0)
Characteristic (continued)	Fluoxetine (n=40)*	Naltrexone (n=40)*
PTSD	0 (0)	0 (0)
Lifetime psychotic disorders	1 (3)	1 (3)
Anorexia	1 (3)	0 (0)
Bulimia	0 (0)	0 (0)
Binge eating		
Mild	2 (5)	0 (0)
Moderate	3 (8)	2 (5)
Severe	0 (0)	0 (0)
Generalised anxiety disorder	2 (5)	3 (8)
Antisocial personality disorder	1 (3)	1 (3)
Neuropsychiatric diagnosis, No. (%)		
ADHD	4 (10)	4 (10)
ADHD+autism	0 (0)	1 (3)
Autism	0 (0)	1 (3)
Treatment with CNS stimulants, No. (%)	4 (10)	2 (5)
Previous substance use disorder, No. (%)	3 (8)	7 (18)
Earlier suicide attempts, No. (%)	4 (10)	4 (10)
No earlier/current comorbidity, No. (%)	15 (38)	14 (35)
Type of problematic sexual behaviours, No. (%)		
Solitary	9 (23)	14 (35)
Non-solitary	5 (10)	2 (5)
Both	27 (68)	24 (60)
Paraphilic interest, No. (%)	18 (45)	14 (35)
Paraphilic disorder, No. (%)	7 (18)	3 (8)
Age of CSBD debut, median (IQR)	18 (14.25–25.00)	19.5 (16.00–25.75)
Years of experienced CSBD problems, median (IQR)	15 (9.25–24.75)	14 (9.25–20.00)
Number of problematic sexual behaviours, median (IQR)	3 (2–3.75)	3 (2.00–4.00)
Measurements:		
HD:CAS, median (IQR)	14 (11–15)	14 (12–17)
MADRS-S, median (IQR)	18.5 (15.25–23.00)	18.5 (13.25–26.75)
AUDIT, median (IQR)	4 (2.25–8.00)	4 (2.00–6.00)
DUDIT, median (IQR)	0 (0–0)	0 (0–0)
GDIT, median (IQR)	0 (0–3)	0 (0–2)
SCS, median (IQR)	30 (26.25–35.75)	31 (26.00–34.00)
HBI-19, mean (SD)	73.57 (12.16)	76.15 (13.11)
ASRS, mean (SD)	29.88 (13.66)	34.05 (12.28)

* Percentages may not total 100 due to rounding.

† According to Kinsey Heterosexual-Homosexual Rating Scale.

ADHD, Attention Deficit/Hyperactivity Disorder; ASRS, Adult ADHD Self-Report Scale; AUDIT, Alcohol Use Disorders Identification Test; CNS, central nervous system; CSBD, compulsive sexual behaviour disorder; DUDIT, Drug Use Disorders Identification Test; GDIT, The Gambling Disorder Identification Test; HBI-19, Hypersexual Behaviour Inventory; HD:CAS, Hypersexual Disorder: Current Assessment Scale; MADRS-S, Montgomery-Åsberg Depression Rating Scale Self-Rating; M.I.N.I, Mini International Neuropsychiatric Interview; PTSD, post-traumatic stress disorder; SCS, Sexual Compulsivity Scale.

Baseline socio-demographic and psychiatric characteristics were generally balanced between groups, with only minor numerical differences in paraphilic disorders and previous substance use disorders (both 10/80, 12.5%). Exploratory baseline analyses revealed no significant differences in baseline CSBD symptom severity or substance use measures between participants with and without previous substance use disorder or paraphilic disorders.

### Symptom outcomes

The ITT analysis indicated that there was no significant overall difference in HD:CAS scores between treatment groups at baseline ($\beta_{2}$= −0.58; p=0.48). HD:CAS scores changed significantly over time in both treatment groups, with significant treatment-by-time interactions for the quadratic (p=0.02) and cubic contrasts (p=0.01). These findings indicate that the pattern of change over time differed between treatment groups (figure 2). However, there were no statistically significant differences between the two drugs at the end of treatment (p=0.96).

**Figure 2:**
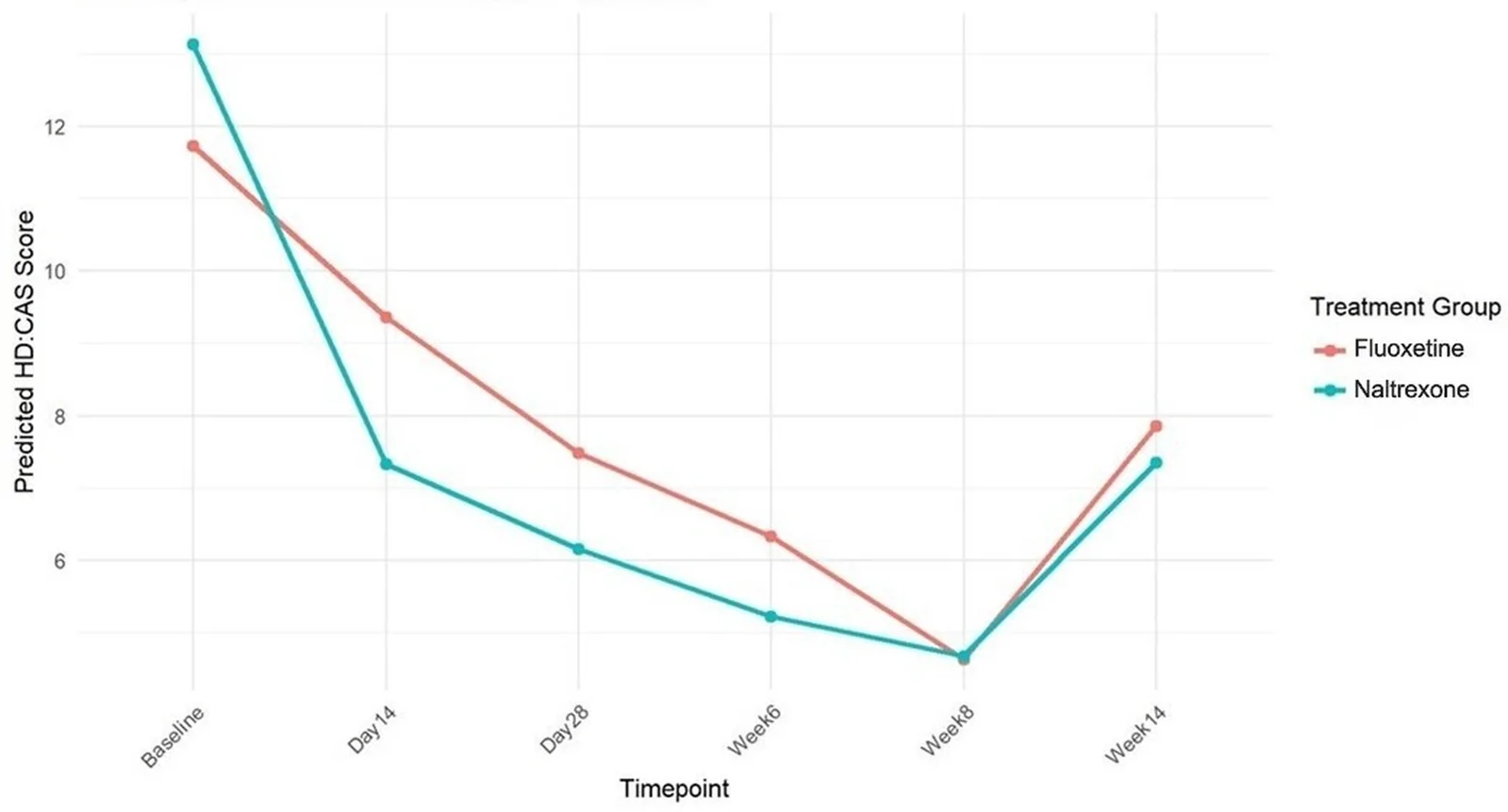
Estimated marginal means of HD:CAS scores over time by treatment group in the ITT analysis. HD:CAS, Hypersexual Disorder: Current Assessment Scale; ITT, intention-to-treat.

Post hoc analyses further indicated distinct within-group patterns in the timing and magnitude of symptom score changes over time. The estimated mean decrease of HD:CAS scores in the fluoxetine group was significant between baseline and week 6 (5.40; p=0.02), and baseline and week 8 (7.10; p=0.001) (table 2). Scores increased again following treatment discontinuation at week 8, but this was not statistically significant (3.23 points; p=0.40) (online supplemental table 3).

**Table 2:** Estimated change in HD:CAS score in the fluoxetine and naltrexone groups from baseline to week 14: ITT analysis

Contrast	Estimated change ITT fluoxetine	P value ITT fluoxetine	Estimated change ITT naltrexone	P value ITT naltrexone
Baseline to day 14	2.375	0.7270	5.800	**<0.0001**
Baseline to day 28	4.250	0.1249	6.975	**<0.0001**
Baseline to week 6	5.400	**0.0194**	7.900	**<0.0001**
Baseline to week 8	7.100	**0.0005**	8.450	**<0.0001**
Baseline to week 14	3.875	0.2029	5.775	**<0.0001**

Bold values indicate statistically significant results (p < 0.05).

HD:CAS, Hypersexual Disorder: Current Assessment Scale; ITT, intention-to-treat.

In the naltrexone group the estimated decrease of HD:CAS scores was significant (p<0.001) between baseline and all later time points (day 14 through week 14). The greatest decrease was observed between baseline and week 6 (7.90 points) and baseline and week 8 (8.45 points) (table 2). A significant increase between week 8 and week 14 (2.68 points; p=0.006) was observed, suggesting a decline in effect after discontinuation (online supplemental table 4).

The PP analysis was broadly consistent with the ITT analysis; however, post hoc pairwise comparisons in the fluoxetine group showed no statistically significant differences between time points. Detailed results are presented in the eResults in the online supplemental file (supplemental figure S2, supplemental tables S5 and S6), including raw mean HD:CAS values at each time point (supplemental tables S7 and S8) and additional CSBD measures (supplemental figures S3–S6, supplemental tables S9–S12). Consistent with the ITT analysis, between-group differences in patterns of change were observed for some, but not all, outcome measures.

### Study retention and adverse effects

Four participants from the fluoxetine group (4/39, 10%) and six from the naltrexone group (6/40, 15%) dropped out (figure 1), with no significant difference between the groups (χ² (1, n=79)=0.40; p=0.53). At least one AE was reported by 37 participants in each treatment group (95% in fluoxetine group and 93% in naltrexone group) (online supplemental table 13). Almost all treatment-related AEs were grade 1 or 2. The most common AEs associated with fluoxetine were decreased libido (28/39, 72%), delayed ejaculation (18/39, 46%) and fatigue (10/39, 26%). For naltrexone, the most prevalent AEs included decreased libido (24/40, 60%), fatigue (23/40, 58%) and nausea (16/40, 40%). Two participants who received fluoxetine (2/39, 5%) discontinued treatment due to grade 3 AE (urticaria and elevated liver enzymes, respectively). Treatment-emergent AEs details are included in online supplemental table 14).

### Treatment adherence

The daily dosage of fluoxetine was 20 mg for n=32 (82%); 40 mg for n=7 (18%). Naltrexone was administered at a daily dosage of 50 mg for n=39 (98%) participants and 25 mg for n=1 (3%). Compliance, measured by a medication count at the end of treatment, was 97.7% (87–102%) for fluoxetine participants and 94.2% (80–103%) for naltrexone participants (>100% indicates taking more medication than prescribed). Mean blood concentrations of combined fluoxetine and its active metabolite norfluoxetine were measured at the end of the treatment (n=26), with a mean level of 640.54 nmol/L (SD=228.91) well within the proposed guideline range of 380–1600 nmol/L when used for the primary indication.^21^ Reasons for missing or unavailable blood collection data included delayed visits, incorrectly handled samples, dropouts or exclusion by Principal Investigator.

### Continuation preferences

Two participants per group experienced increased CSBD symptoms post-treatment and resumed their assigned medication at their own request before the end of the trial at week 14. Including these, 13 participants in the fluoxetine group (13/33, 39%) and 14 (14/34, 41%) in the naltrexone group wished to resume their assigned medication at the end of the trial, while nine participants in each group (fluoxetine 9/33, 27%, naltrexone 9/34, 27%) did not and 11 were unsure (fluoxetine 11/33, 33%, naltrexone 11/34, 32%). The wish to resume treatment did not differ significantly between the groups, χ² (2, n=67)=0.02; p=0.99.

## Discussion

In this RCT involving participants with CSBD, naltrexone was not statistically superior to fluoxetine after 2 months of treatment. However, significant treatment-by-time interactions indicated differences in the pattern of symptom change over time across treatment groups. Exploratory post hoc analyses suggested different symptom trajectories, with earlier within-group symptom reduction in the naltrexone group (within 2 weeks) than in the fluoxetine group (significant at week 6). As the trial was not designed to formally test differences in onset of action between treatments, this finding should be considered exploratory. PP post hoc within-group analyses did not confirm this for fluoxetine, potentially influenced by early discontinuation (including two grade 3 AEs), reducing power. A significant post-treatment increase in HD:CAS score was observed in the naltrexone group, indicating that symptom reduction may not be sustained after discontinuation; fluoxetine results were less consistent, possibly reflecting differences in the pharmacodynamic properties of the treatments.^7 22^

The absence of significant differences between naltrexone and fluoxetine in our trial is consistent with the 20 weeks RCT by Lew-Starowicz *et al* comparing naltrexone, paroxetine and placebo.^10^ Although the groups did not differ significantly on the primary outcome in either trial, higher satisfaction ratings in the active-treatment groups in the trial by Lew-Starowicz *et al* indicate that existing measures used in CSBD research, including this trial, may not capture all relevant treatment dimensions.

Although AEs were reported by most participants, they were mainly mild and adherence remained high, suggesting good tolerability. Nevertheless, two cases of serious AEs in the fluoxetine group led to discontinuation, underscoring the importance of routine safety monitoring. Although potentially desirable in CSBD, decreased libido was recorded as an AE, which was further the most commonly reported AE. Prior RCT data of citalopram in men with CSBD suggest sexual AEs may mediate symptom improvement,^11^ complicating the interpretation of treatment mechanisms.

Most participants declined to resume treatment, consistent with our prior finding.^9^ Clinical observations indicate that this may reflect sufficient symptom improvement, perceived lack of efficacy, dissatisfaction due to AEs or a wish for other treatment strategies such as psychotherapy. In summary, even though quick symptom relief is desirable and the observed pattern of symptom change may suggest potential advantages of naltrexone, clinical decision-making should consider individualised assessment of adverse event profiles, patient preferences and ease of administration.

### Strengths and limitations

Key strengths of our trial include randomisation, frequent assessments to track symptom change and controlling for interference factors such as ongoing psychotherapy. Furthermore, participants were thoroughly assessed by specialists to accurately identify other issues, such as moral incongruence, resulting in a well-characterised study sample. This is crucial as not meeting diagnostic criteria for CSBD was the most common reason for exclusion.

This trial has several limitations. The main outcome measure is not validated and was developed for hypersexual disorder; additional measures were therefore included. The trial was not blinded and lacked a placebo arm due to cost in this non-industry-sponsored trial, leaving anticipation effects uncontrolled for. More participants with past substance use disorder were randomised to receive naltrexone, and more participants with paraphilic disorder were randomised to receive fluoxetine; however, these subgroups were relatively small in absolute numbers and did not differ on symptom measures. The finding that 40% of participants reported paraphilic interests aligns with our clinical experience and further underscores the need for early identification of problematic sexual behaviours and interventions to address potential risk factors for illegal behaviours. Future studies on CSBD should also consider measuring craving as a predictor of response patterns. The 14-week duration is short, partly limited by pandemic-related feasibility constraints. Future studies should extend follow-up periods, examine higher dosages of the drugs or stepped-care approaches, especially among treatment-resistant patients. Future research should also aim to examine populations underrepresented in this trial, especially women and younger individuals, as CSBD typically begins in early adulthood and our sample reported an average of 15 years of symptoms before seeking treatment. Finally, identifying predictors of treatment response or subgroups with distinct clinical response profiles^23^ will help to optimise individualised treatment strategies in CSBD.

## Clinical implications

Although no overall superiority of naltrexone over fluoxetine was observed, participants in the two treatment groups showed different patterns of symptom improvement over time. Exploratory within-group analyses showed significant symptom improvement in the naltrexone group from early treatment onward across outcome measures, including measures beyond the unvalidated primary outcome. If replicated, these findings may support further investigation of naltrexone as a potential short-term adjunct to psychotherapy during the initial stabilisation phase of treatment.

A significant post-treatment worsening in symptoms was observed in the naltrexone group after treatment discontinuation; fluoxetine results were less consistent. Compliance and tolerability were in general equivalent between treatments although the drugs presented different AE profiles.

In summary, clinical decisions may benefit from an individualised assessment that considers patient characteristics, treatment goals and preferences, alongside the potential differences in response between the drugs and differing adverse effect profiles.

## Supplementary material

10.1136/bmjment-2026-302953 online supplemental figure 1

## Data Availability

Data are available upon reasonable request.
