# Supplementary material for: Randomised controlled trial of fluoxetine versus naltrexone in compulsive sexual behaviour disorder
Source: BMJ Ment Health. 2026 Oct 6;29(1):e302953. doi: 10.1136/bmjment-2026-302953 (PMC13646924; doi:10.1136/bmjment-2026-302953)
Supplement: Supplementary file 1 [file bmjment-29-1-s001.pdf]

# A randomised controlled trial of fluoxetine versus naltrexone in compulsive sexual behaviour disorder- Supplementary material

## Contents


**Supplemental table 1. Inclusion and exclusion criteria** ....................2

**eMethods** .................... 3

**Hypersexual Disorder: Current Assessment Scale (HD:CAS)**.................... 7

**Supplemental figure 1. Schedule of enrolment, interventions, and assessments** .....10

**Supplemental table 2. Baseline characteristics of dropout participants, before onsite assessment (n=20)**....................12

**Supplemental table 3. Estimated change in HD:CAS score in the fluoxetine group over time: ITT analysis** ....................14

**Supplemental table 4. Estimated change in HD:CAS score in the naltrexone group over time: ITT analysis** ....................15

**eResults**....................16

**Supplemental figure 2. Estimated marginal means of HD:CAS scores over time by treatment group in the PP analysis** ....................16

**Supplemental table 5. Estimated change in HD:CAS score in the fluoxetine group over time: PP analysis**....................16

**Supplemental table 6. Estimated change in HD:CAS score in the naltrexone group over time: PP analysis**....................17

**Supplemental table 7. Changes in raw mean values of HD:CAS over time by treatment group, ITT dataset**....................18

**Supplemental table 8. Changes in raw mean values of HD:CAS over time by treatment group, PP dataset** ....................19

**Supplemental figure 3. HBI interaction effects ITT**....................20

**Supplemental figure 4. HBI interaction effects PP** ....................20

**Supplemental table 9. Estimated change in HBI score over time in the fluoxetine group: ITT and PP analysis** ....................21

**Supplemental table 10. Estimated change in HBI score in the naltrexone group over time: ITT and PP analysis**....................21

**Supplemental figure 5. SCS interaction effects ITT**....................22

**Supplemental figure 6. SCS interaction effects PP** ....................23

**Suplemental table 11. Estimated change in SCS score in the fluoxetine group over time: ITT and PP analysis**....................23

**Supplemental table 12. Estimated change in SCS score in the naltrexone group over time: ITT and PP analysis**....................24

**Supplemental table 13. Summary of adverse events (AE) (occurring in ≥5% of patients) by MedDRA system** ........ 25

**Supplemental table 14. All treatment-emerged adverse events reported by patients (N=79), by MedDRA system organ class and preferred term** ........ 26

**eReferences** ........ 28

## Supplemental table 1. Inclusion and exclusion criteria

| | |
|---|---|
| **Inclusion criteria** | |
| 1. | Meet criteria for compulsive sexual behaviour disorder according to ICD-11 and criteria for hypersexual disorder as originally proposed for inclusion in DSM-5. |
| 2. | Aged 18-65 years. |
| 3. | Able to read and communicate in Swedish and have internet access. |
| 4. | Agree to participate in all study visits including providing blood and urine samples. |
| 5. | Signed informed consent form. |
| 6. | For fertile women: the use of a safe method of contraception during the entire study protocol. |
| **Exclusion criteria** | |
| 7. | Signs of hepatitis, elevated liver enzymes (>3 times over reference), or a history of liver failure. |
| 8. | eGFR <60 ml/min, signs or history of acute kidney failure. |
| 9. | fP-glucose ≥7.0 mmol/l (126 mg/dl) |
| 10. | History of heart disease such as angina pectoris, heart failure, or heart attack. |
| 11. | Other serious physical illness including diabetes mellitus, epilepsy, or known ocular hypertension. |
| 12. | Treatment in the past month (≥1 dose) with opioids or benzodiazepines. |
| 13. | Treatment in the past month with oral anticoagulants such as warfarin. Intermittent treatment (max. 15 doses per week) with NSAID (eg, ibuprofen) is tolerated. |
| 14. | Treatment with tamoxifen. |
| 15. | Self-reported use of recreational drugs in the past month or positive drug verification analysis. |
| 16. | Alcohol dependence or risk consumption (>14 units of alcohol per week for men, >9 for women, where 1 unit=12 g alcohol) in the past month. |
| 17. | Severe psychiatric disorder requiring immediate treatment such as current psychotic disorder or severe depression. |
| 18. | Bipolar disorder or history of hypomania. |
| 19. | Ongoing treatment with naltrexone or SSRI, or previous hypersensitivity reaction to either. |
| 20. | Change of concurrent medication or dosage in the past three months regarding antidepressants, ADHD medication, mood stabilisers, antipsychotics, cortisone, testosterone, or dopamine precursors. Smaller adjustments may in some cases be acceptable (as assessed by trial psychiatrist). |
| 21. | Ongoing pharmacological treatment with contraindicated substances (eg, tamoxifen, metoprolol). |
| 22. | Pregnancy and/or breastfeeding. |
| 23. | Mental health condition that could negatively influence either the participant’s health or the scientific aspects of the study. High risk for committing sexual offense is included. |
| 24. | Ongoing psychotherapeutic treatment. |
| 25. | Participation in other studies outside ANOVA. |

*Abbreviations:* ADHD=attention-deficit/hyperactivity disorder; NSAID=nonsteroidal anti-inflammatory drug; DSM-5=Diagnostic and Statistical Manual of Mental Disorders, Fifth Edition.

## eMethods

Study design

This is an academically initiated, open-label, randomised controlled parallel-group trial. The participants were block randomised and allocated 1:1 to receive either fluoxetine (n=40) or naltrexone (n=40) for 8 weeks (primary endpoint), followed by 6 weeks without treatment.

One minor protocol change was implemented[1] after trial commencement regarding an update on data management according to General Data Protection Regulation (GDPR) (17 December 2021).

Study setting and recruitment

The study was conducted at ANOVA, a centre for sexual medicine, andrology, and trans medicine at Karolinska University Hospital, Stockholm, Sweden. Help-seeking individuals with symptoms consistent with CSBD and likely to meet eligibility criteria (Supplementary material table 1) received information about the study through PrevenTell, a Swedish national helpline for problematic sexuality operated by the ANOVA clinic.[2] Potential participants were invited to log into a secure web-based platform to provide online consent and complete questionnaires covering sociodemographic, eligibility, and sexual factors (further described below). The study was also advertised on the websites of the Karolinska Insitutet, ANOVA, and PrevenTell, where potential participants could indicate interest for the study and access the web-based platform. An additional telephone screening regarding compulsive sexual behaviour as well as inclusion and exclusion criteria was conducted by the study team after the participant had filled out the online questionnaires. Participants likely to meet eligibility criteria were subsequently invited to the clinic for further information and evaluation. A written informed consent was collected at the baseline visit. The visits at the clinic and the drugs were free of charge. No compensation was offered. The trial ended with the inclusion of the last participant.

Eligibility criteria

The trial includes individuals meeting the ICD-11 criteria for CSBD within the age range 18 to 66 years. Individuals with severe physical illness, mental health condition, or substance use disorders were excluded. Participants were not eligible for inclusion if they had any concomitant psychotherapy or changes to relevant medication during the preceding three months. For a detailed account of screening procedures and assessments and the complete eligibility criteria, see Supplementary figure 1, Supplementary table 1, and the study protocol.[1] The screening visit at the ANOVA clinic included two consecutive evaluations: one from a board-certified psychiatrist and one from a clinical psychologist. Blood samples were collected (for eg, hormone, metabolic, liver, and electrolyte panel). Urine samples were screened for recreational drug use (eg, amphetamine, benzodiazepines, cannabis, cocaine, and opioids). The psychiatrist obtained information on the individual's sexual and psychiatric background as well as conducted the Mini International Neuropsychiatric Interview 7.0.0,[3] and Columbia Suicide Severity Rating Scale.[4] The psychiatrist also obtained the individual's medical history and performed a physical examination (ie, blood pressure, heart and pulmonary auscultation). The psychologist conducted a structured interview focusing on sexual behaviours. During the clinical assessments, participants were systematically evaluated for paraphilic interests or paraphilic disorders in accordance with the Diagnostic and Statistical Manual of Mental Disorders, Fifth Edition (DSM-5) criteria. To control for bias, the psychiatrist and the psychologist then independently assessed and confirmed fulfilment of the eligibility criteria. The participant was not included in the event of discrepant assessments.

Randomisation

The research nurse performed the randomisation using opaque, sealed envelopes provided by an independent clinical trial support unit, the Karolinska Trial Alliance. The allocation sequence was concealed to the research team and participants. All study drugs were provided by the Karolinska University Hospital pharmacy and given to the enrolled participants by the research nurse. Due to the clinic's geographic location and time delays for laboratory services during the covid-19 pandemic, the full blood test results were only received the next day. As many participants had a long travel route to the clinic, it was decided to carry out randomisation and provide medication before the blood test results arrived. Participants were contacted the next day and instructed to start treatment if blood test results were adequate, as deemed by the study psychiatrist. Participants were given a written information sheet about their allocated drug and information on study procedures.

Interventions

The Swedish Dental and Pharmaceutical Benefits Agency, a national government agency, decides

which drugs are to be subsidised and recommends specific products based on factors such as price and availability. The manufacturers of naltrexone and fluoxetine used in this study were those recommended by the agency at the time of submission of the study protocol. The pharmaceutical industry was not involved in any aspect of the study.

Participants allocated to fluoxetine started with an initial dose of 20 mg/day which was augmented to 40 mg/day after 4 weeks if the medication was tolerated but symptom reduction was not satisfactory. At week 7 these participants reverted to 20 mg/day for a washout period prior to the end of treatment. The initial dose for those assigned naltrexone was 25 mg/day, which was increased to 50 mg/day after 3-5 days if tolerated.

Participants assessed their own symptoms by completing online questionnaires at regular intervals (Supplementary figure 1). Reminder messages were sent to their mobile phones for three consecutive days following activation of the assessment. New blood samples were collected for safety reasons at week 4, followed by a telephone consultation with the study psychiatrist to assess adverse events and psychiatric symptoms.

At the end of treatment, blood samples were collected and participants had a consultation with the psychiatrist to assess current symptoms of CSBD and psychiatric wellbeing. Tolerability was assessed throughout the study period and the participant was able to report adverse effects through open-ended questions on the web platform or during contact with the study psychiatrist. All adverse events were noted in a paper log (at least one episode/event) with an assessment of severity (mild/moderate/severe), evaluation of causality related to treatment (likely/possibly/unlikely), and any actions taken in response. Serious adverse events and suspected unexpected serious adverse reactions were managed and reported in accordance with the regulatory requirements of the Swedish Medical Products Agency. The participant also filled in the Udvalg for Kliniske Undersogelser (UKU) side effect rating scale at the primary endpoint to systematically assess harms. The psychiatrist confirmed the UKU responses and did a summative evaluation of the adverse effects including those registered in the paper log. Six weeks after the end of treatment, the participants filled in follow-up questionnaires and had a final consultation with the study psychiatrist.

Compliance and concomitant care

Participants could discontinue study participation at any time without providing a reason. Participants who missed ≥3 doses in a row were considered to have discontinued treatment. Other reasons for termination of treatment included severe adverse reactions, severe mental health condition that needed to be prioritised, the need for or initiation of treatment with non-compatible drugs, use of recreational drugs, conditions where the safety of the participant or others could not be guaranteed, start of psychotherapy, or pregnancy.

At the end of treatment, the participants who received fluoxetine provided a blood sample to assess concentrations of fluoxetine and norfluoxetine. Due to the covid-19 pandemic, the Karolinska research laboratory was unfortunately unable to prioritise the corresponding analysis of naltrexone concentration. Participants were encouraged to return any unused tablets for a medication count.

## Measures

*Primary outcome measures*

The Hypersexual Disorder: Current Assessment Scale (HD:CAS)[5] measures symptom severity during the preceding 2 weeks according to the conceptualisation of hypersexual disorder suggested for DSM-5.[6] A corresponding scale following the ICD-11 criteria was not developed at the time of planning the study. HD:CAS has been used in prior studies by our research team for the evaluation of both pharmacological and therapeutic interventions for CSBD[7,8,9] and has been deemed sensitive to detect symptom changes. The scale comprises two parts; the first part covers behaviours related to CSBD (eg, masturbation and the use of pornography). In the second part, the participant quantifies the behaviours and evaluates perceived control as well as risk and harm in relation to these behaviours. The six items in part two are scored on a Likert scale (0-4 points) with a total score of 0 to 24 points. As the primary scale is not validated, two additional measures of CSBD were used. The Hypersexual Behaviour Inventory (HBI-19)[10] is a validated 19-item scale assessing hypersexual disorder based on the proposed criteria for DSM-5. Each item is rated on a 5-point Likert scale (1-5), resulting in a total score of 19 to 95, with a threshold of ≥53 points suggested as an indicator of CSBD.

Sexual Compulsivity Scale (SCS)[11] measures sexual compulsivity with ten items, on a 4-point scale (1-4), with a total score of 10 to 40.

*Sociodemographic and clinical characteristics measures*

Sexual orientation was assessed on the Kinsey Heterosexual - Homosexual Rating Scale.[12] The scale ranges from 0-6, where 0 means exclusively heterosexual, 6 means exclusively homosexual, and 1-5 signify varying levels of interest for either sex.

The Montgomery-Åsberg Depression Rating Scale - self-rating version (MADRS-S) assesses changes in depressive symptoms using nine items, with a total score of 0 to 54.[13]

The Adult ADHD Self-Report Scale (ASRS) evaluates ADHD symptoms with 18 items, with a total score of 0 to 72.[14]

The Alcohol Use Disorders Identification Test (AUDIT) assesses alcohol consumption and related problems with ten items, with a total score of 0-40; a score of ≥8 indicates harmful use in men and ≥6 harmful use in women.[15,16]

The Drug Use Disorders Identification Test (DUDIT) assesses the use of drugs and related problems with 11 items, with a total score of 0 to 44.[17]

The Gambling Disorder Identification Test (GDIT) assesses pathological gambling with 14 items, with a total score of 0 to 62.[18]

The Hypersexual Disorder Screening Inventory (HDSI) assesses hypersexuality and consists of two sections, along with a set list of questions about problematic, normophilic behaviours over the preceding six months, including masturbation, pornography use, sex with consenting adults, cybersex, phone sex, strip club visits, and other behaviours, with an option to specify details.[5] For this study, "other" behaviours were categorised by the study leads into existing categories. To identify potential subgroups of behaviours, these were categorised as solitary (masturbation and pornography use) or non-solitary (remaining indicators).

*Tolerability and treatment satisfaction measures (secondary outcomes)*

Tolerability was evaluated non-systematically thorough all time points and systematically with the UKU side effect rating scale at week 8.[19] The validated UKU self-rating scale covers 48 symptoms to capture perceived adverse events. Harms classified as having a possible or probable causal relationship with the intervention (as described under interventions) were grouped and coded according to the Medical Dictionary for Regulatory Activities (MedDRA, version 27.1) system organ class and preferred terms. The severity of treatment-emergent adverse events (AE) was assessed using the US National Cancer Institute Common Terminology Criteria for Adverse Events version 5.0. Other measures of tolerability and treatment satisfaction included number of dropouts, drug compliance at the end of treatment, as well as participants' wish to resume pharmacological treatment at the end of the study (with the options: yes/no/do not know).

Sample size and statistical methods

The power calculation was based on the variance components of HD:CAS from a cognitive behavioural therapy study conducted by our research team[8] and specified in the study protocol.[1] A sample size of 68 would be required to detect a clinically significant difference with a power of 80% and a significance level of 0.05. To account for dropouts, 80 participants were planned to be randomised.

Baseline characteristics were analysed using descriptive statistics and presented as frequencies and percentages. The Shapiro-Wilk test was used to assess normality. Non-normally distributed variables were reported as median (Mdn) and interquartile range (IQR). For normally distributed data, mean (M), standard deviation (SD), and range were presented.

The intention-to-treat (ITT) analysis included all randomised participants (n=40 per group). For the per protocol (PP) analyses, only those who completed treatment and assessments at week 8 were included (n=66, 32 in the fluoxetine group and 34 in the naltrexone group).

Linear mixed models (LMM) were used for the ITT and PP analyses of the treatment effects. The model was expressed as follows: $\mathrm{yij} = \beta 0 + \beta 1(\mathrm{Time}) + \beta 2(\mathrm{Treatment}) + \beta 3(\mathrm{Time} \times \mathrm{Treatment}) + \mathrm{bi} + \mathcal{E}\mathrm{ij}$.

Time was defined as a categorical variable, with each time point representing a measurement occasion. The baseline measurements served as the reference; to account for individual differences, each participant's baseline measurements were considered as random intercepts. The interaction variable was used to analyse changes over time across treatment. Post hoc tests to compare means used the Tukey method. No imputation was performed to handle missing values as linear mixed models are not sensitive to missing data, given the assumption that data are missing at random.

Comparisons of the number of dropouts and individuals who wished to resume treatment were explored with chi-square tests. All statistical analyses were performed using R version 4.4.1 and SPSS version 29.0 (IBM Corp., Armonk, NY, USA). The statistician who performed the data analysis was blinded to the group allocation. No interim analyses were conducted.

Monitoring and data management

An independent trial investigator from the Karolinska Trial Alliance regularly controlled and quality assured the study procedures to ensure adherence to good clinical practice. This included confirmation of collected data and verification of drug handling procedures. The web-based platform that contains participants' data is stored securely on servers with restricted access.

Ethics

The Swedish Ethical Review Authority and the Swedish Medical Products Agency approved the study (ref. no. 2020-02069 and ref. no. 5.1-2020-48282). The trial was pre-registered (Eudra-CT no. 2019-004255-36) and accessible at https://www.clinicaltrialsregister.eu. The study procedures were carried out in accordance with the Declaration of Helsinki.

Patient and public involvement

The patients and public were not involved in the design of the study. All patients were, however, aware of the trial objectives during the study recruitment.

## Hypersexual Disorder: Current Assessment Scale (HD:CAS)

Your name: Identification #:

Date:

**A.1. Place an "X" mark on the line to the left of each different kind of sexual behavior that you think caused you to have significant problems <u>during the past 2 weeks:</u>**

____ Masturbation, either by itself or during other sexual activities

____ Pornography (some examples include: Internet video, images and webcasts, magazines, DVDs/videos, X-rated TV and films)

____ Sexual Behavior with Consenting Adults (direct contact) (some examples include: use of escort services, prostitutes, repeated "one–night stands", anonymous brief sexual encounters, repeated affairs, massage parlor visits that include sex.)

____ Cybersex activities (some examples include: Internet related sexual talk, sexual behavior associated with web-cams, other 'virtual' sexual behaviors)

____ Telephone Sex

____ Strip Clubs

____ Other sexual behaviors: (please specify________________)

**The next series of questions concern your problematic sexual behavior (marked above in A.1) during the past 2 weeks.**

A.2

During the past 2 weeks (14 days), how many times per week did you have an orgasm (climax) while engaging in all of your problematic sexual behavior ?

0. none

1. up to 1 time per week

2. 2-4 times per week

3. 5-8 times per week

4. 9 or more times per week

A.3

During the past 2 weeks (14 days), on average, how much time per day was consumed by all of your problematic sexual fantasies, sexual urges or sexual behavior?

0. no time

1. up to 15 minutes in the average day

2. between 16 minutes and 1 hour in the average day

3. between 1 to 3 hours in the average day

4. more than 3 hours in the average day

A.4

During the past 2 weeks, how frequently did you use sexual fantasies and sexual behavior to cope with difficult feelings (for example, worry, sadness, boredom, frustration, guilt, or shame)?

0. never

1. only occasionally when I engaged in sexual behavior

2. some of the times that I engaged in sexual behavior

3. almost every time I engaged in sexual behavior

4. every time I engaged in sexual behavior

A.5

During the past 2 weeks, how frequently did you use sexual fantasies and sexual behavior to avoid, put off, or cope with stresses and other difficult problems or responsibilities?

0. never

1. only occasionally when I engaged in sexual behavior

2. some of the times that I engaged in sexual behavior

3. almost every time that I engaged in sexual behavior

4. every time that I engaged in sexual behavior

Please continue with questions on page 3.

A.6

During the past 2 weeks, on the average how much control did you feel you had over sexual fantasies,urges and behavior?

0. in control all of the time

1. in control almost all the time

2. in control some of the time

3. rarely in control

4. never in control

A.7

During the past 2 weeks, how many times per week did you engage in sexual behavior that you would consider risky, harmful or even dangerous for yourself, your partner(s) or another significant relationship?

0. none

1. up to 1 time per week

2. 2-4 times per week

3. 5-8 times per week

4. 9 or more times per week

*Prepared by the Paraphilias Subworkgroup of the Work Group on Sexual and Gender Identity Disorders for DSM-V, July 15, 2009. Modified for 12.01.2009 by Martin P. Kafka, MD. Included with permission from Martin P. Kafka, MD, personal communication, August 14, 2026.*

## Supplemental figure 1. Schedule of enrolment, interventions, and assessments

| | Pre-allocation | | Allocation | Treatment period | | | | | | | | Follow-up |
|---|---|---|---|---|---|---|---|---|---|---|---|---|
| **TIMEPOINT** | **$-t_2$** | **$-t_1$** | **0** | **$t_1$ Day 0** | **$t_2$ Day 3-5** | **$t_3$ 1 wk** | **$t_4$ 2 wk** | **$t_5$ 3 wk** | **$t_6$ 4 wk** | **$t_7$ 6 wk** | **$t_8$ 8 wk** | **$t_9$ 14wk** |
| **ENROLMENT:** | | | | | | | | | | | | |
| ***Screening by telephone*** | X | | | | | | | | | | | |
| ***Evaluation by Psychiatrist (on site)*** | | X | | | | | | | | | X | |
| ***Evaluation by Psychiatrist (telephone)*** | | | | | X | | | | X | | | X |
| ***Evaluation by psychologist (on site)*** | | X | | | | | | | | | | |
| ***Physical examination*** | | X | | | | | | | | | | |
| ***Urine and blood samples [a]*** | | X | | | | | | | X | | X | |
| ***Specific research samples [b]*** | | X | | | | | | | | | X | |
| ***Eligibility screen*** | X | X | | | | | | | | | | |
| ***Eligibility confirmation*** | | | | X | | | | | | | | |
| ***Informed consent*** | X | X | | | | | | | | | | |
| ***Allocation*** | | | X | | | | | | | | | |
| **INTERVENTIONS:** | | | | | | | | | | | | |
| ***Fluoxetine*** | | | | | — | — | — | — | — | — | — | |
| ***Naltrexone*** | | | | | — | — | — | — | — | — | — | |
| ***Dosage adjustment (if warranted)*** | | | | | Naltr-exone | | | | Fluox-etine | | | |
| **ASSESSMENTS:** | | | | | | | | | | | | |
| ***Demographic information*** | X | | | | | | | | | | | |
| ***Structured interview [c]*** | | X | | | | | | | | | | |
| ***Self-report screening battery [d]*** | X | | | | | | | | | | | |
| ***Self-report outcome battery 1 [e]*** | | | | | | X | X | X | X | X | | |

| | | | | | | | | | | | | |
|---|---|---|---|---|---|---|---|---|---|---|---|---|
| ***Self-report outcome battery 2*** [f] | | | | | | | | | | | X | X |
| ***HD: CAS (main outcome)*** | X | | | | | | X | | X | | X | X |
| ***UKU*** | | | | | | | | | | | X | |
| ***C-SSRS*** | | | | | X | | | | X | | X | X |
| ***Pill count*** | | | | | | | | | | | X | |
| ***AE, SAE, and SUSAR*** | | | | | ◄— | — | — | — | — | — | — | —► |

Abbreviations: wk, week; HD:CAS, Hypersexual Disorder: Current Assessment Scale; UKU, Udvalg for Kliniske Undersogelser side effect rating scale; C-SSRS, Columbia Suicide Severity Rating Scale; AE, Adverse Event; SAE, Serious Adverse Event; SUSAR, Suspected Unexpected Serious Adverse Reaction.
a Samples at -t1: Full blood cell count, Metabolic panel (fP-Glucose, B-HbA1c, P-HDL-Cholesterol, P-Cholesterol, P-LDL-Cholesterol, P-Triglyceride), Liver panel (P-ASAT, P-ALAT, P-GT, P-ALP, P-Albumin), Electrolyte panel (P-Sodium, P-Potassium, P-Creatinine, P-Calcium), Hormone panel (S-TSH, S-T4, S-LH, S-FSH, S-SHBG, S-Testosterone), Urine screening for substance use (amphetamine, benzodiazepines, buprenorphine, cannabis, cocaine, fentanyl, methadone, oxycodone, tramadol, and other opioids), U-Pregnancy test for assigned women at birth. Samples at week 4: Full blood count, P-Glucose, Electrolyte Panel (P-Sodium, P-Potassium, P-Creatinine), Liver panel (P-ASAT, P-ALAT, P-GT, P-ALP), U-Pregnancy test for assigned women at birth.
Samples at week 8: Full blood count, B-HbA1c, Electrolyte Panel (P-Sodium, P-Potassium, P-Creatinine), Liver panel (P-ASAT, P-ALAT, P-GT, P-ALP). U-Pregnancy test for assigned women at birth, P-Fluoxetine or U-Naltrexone depending on randomization.
b One 5-10 ml blood collection tube for DNA extraction (-t1 only), and 10 ml for storage in Biobank.
c Interview by psychiatrist at -t2 (includes the Mini International Neuropsychiatric Interview 7.0.0; Interview by psychologist at -t2
d Self-report screening battery includes: The Hypersexual Disorder Screening Inventory; The Hypersexual Behavior Inventory; Sexual Compulsivity Scale; Self-rating sexual interests; International Index of Erectile Function; Montgomery-Åsberg Depression Rating Scale – Self rating; Childhood Trauma Questionnaire – Short Form; Karolinska Interpersonal Violence Scale; Barratt Impulsiveness Scale; Adult ADHD Self-Report Scale; Alcohol Use Disorder Identification Test; Drug Use Disorder Identification Test; Gambling Disorder Identification Test (G-DIT).
e Self-report outcome battery 1 includes: Hypersexual Behavior Inventory; Sexual Compulsivity Scale; Montgomery-Åsberg Depression Rating Scale – Self rating (only final question on suicidal thoughts).
f Self-report outcome battery 2 includes: Hypersexual Behavior Inventory; Sexual Compulsivity Scale; Montgomery-Åsberg Depression Rating Scale – Self rating; Alcohol Use Disorder Identification Test. Post treatment also a question of wanting to resume pharmacological treatment

**Supplemental table 2. Baseline characteristics of dropout participants, before onsite assessment (n=20)**

| | |
|---|---|
| Age in years, M (*SD*)<br>*Range* | 36.15 (*7.34*)<br>*22-49* |
| Gender | |
| *Man* | 19 (95%) |
| *Woman* | 0 (0%) |
| *Other* | 1 (5%) |
| Place of birth | |
| *Sweden* | 18 (90%) |
| *Other country* | 2 (10%) |
| Biological parent(s) born abroad | |
| *Yes* | 6 (30%) |
| *No* | 14 (70%) |
| Ever had steady relationship (at least one month) | |
| *Yes* | 18 (90%) |
| *No* | 2 (10%) |
| Steady relationship past month | |
| *Yes* | 12 (66.7%) |
| *No* | 6 (33.3%) |
| Living situation, last month | |
| *Living alone* | 9 (45%) |
| *Living with spouse/partner(s)* | 11 (55%) |
| *Living with parents* | 0 (0%) |
| *Living with other adults* | 0 (0%) |
| Children living at home | |
| *Yes* | 8 (40%) |
| *No* | 12 (60%) |
| Occupation | |
| *Employed/working* | 15 (75%) |
| *Unemployed* | 1 (5%) |
| *Studying* | 2 (10%) |
| *Other* | 2 (10%) |
| Sexual orientation* | |
| *Heterosexual* | 17 (85%) |
| *1* | 1 (5%) |
| *2* | 0 (0%) |
| *3* | 1 (5%) |
| *4* | 0 (0%) |
| *5* | 1 (5%) |
| *Homosexual* | 0 (0%) |
| *Other* | 0 (0%) |
| **Mental health and psychiatric comorbidities** | |
| Measurements: | |
| HD:CAS; Mdn (IQR) | Median (IQR) 13 (9.25-15) |
| MADRS-S, Mdn (IQR) | Median (IQR) 15.50 (12.25-21.50) |
| ASRS, M (range; *SD*) | 32.75 (16-54; 10.48) |
| AUDIT, Mdn (IQR) | 3.50 (0.25-6.00) |
| DUDIT, Mdn (IQR) | 0 (0-0) |
| GDIT, Mdn (IQR) | 0 (0-2) |
| SCS, Mdn (IQR) | 29.50 (25-33.50) |
| HBI-19, M (range; *SD*) | 69.90 (41-95; 14.80) |
| Number of problematic behaviours, Mdn (IQR) | 2.5 (2-3.75) |
| Type of problematic behaviours | |
| *Solitary* | 4 (20%) |
| *Non-solitary* | 1 (5%) |
| *Both* | 13 (65%) |

| | |
|---|---|
| *None* | 2 (10%) |

Data are n (%), mean (range, SD) or median (IQR).
*Abbreviations:* HD:CAS=The Hypersexual Disorder: Current Assessment Scale; median=Mdn; MADRS-S=Montgomery-Åsberg Depression Rating Scale Self-Rating; mean=M; ASRS=Adult ADHD Self−Report Scale; AUDIT=Alcohol Use Disorders Identification Test; DUDIT=Drug Use Disorders Identification Test; GDIT=The Gambling Disorder Identification Test; SCS=Sexual Compulsivity Scale; HBI-19=Hypersexual Behaviour Inventory.
* According to Kinsey Heterosexual−Homosexual Rating Scale.

**Supplemental table 3. Estimated change in HD:CAS score in the fluoxetine group over time: ITT analysis**

| Contrast | Estimated change ITT | p-value ITT |
|---|---|---|
| Baseline to Day 14 | 2.375 | 0.7270 |
| Baseline to Day 28 | 4.250 | 0.1249 |
| Baseline to Week 6 | 5.400 | **0.0194** |
| Baseline to Week 8 | 7.100 | **0.0005** |
| Baseline to Week 14 | 3.875 | 0.2029 |
| Day 14 to Day 28 | 1.875 | 0.8790 |
| Day 14 to Week 6 | 3.025 | 0.4777 |
| Day 14 to Week 8 | 4.725 | 0.0619 |
| Day 14 to Week 14 | 1.500 | 0.9501 |
| Day 28 to Week 6 | 1.150 | 0.9843 |
| Day 28 to Week 8 | 2.850 | 0.5457 |
| Day 28 to Week 14 | -0.375 | 0.9999 |
| Week 6 to Week 8 | 1.700 | 0.9171 |
| Week 6 to Week 14 | -1.525 | 0.9466 |
| Week 8 to Week 14 | -3.225 | 0.4029 |

**Supplemental table 4. Estimated change in HD:CAS score in the naltrexone group over time: ITT analysis**

| Contrast | Estimated change ITT | p-value ITT |
|---|---|---|
| Baseline to Day 14 | 5.800 | **<0.0001** |
| Baseline to Day 28 | 6.975 | **<0.0001** |
| Baseline to Week 6 | 7.900 | **<0.0001** |
| Baseline to Week 8 | 8.450 | **<0.0001** |
| Baseline to Week 14 | 5.775 | **<0.0001** |
| Day 14 to Day 28 | 1.175 | 0.6327 |
| Day 14 to Week 6 | 2.100 | 0.0648 |
| Day 14 to Week 8 | 2.650 | **0.0070** |
| Day 14 to Week 14 | -0.025 | 1.0000 |
| Day 28 to Week 6 | 0.925 | 0.8273 |
| Day 28 to Week 8 | 1.475 | 0.3767 |
| Day 28 to Week 14 | -1.200 | 0.6110 |
| Week 6 to Week 8 | 0.550 | 0.9788 |
| Week 6 to Week 14 | -2.125 | 0.0593 |
| Week 8 to Week 14 | -2.675 | **0.0062** |

**eResults**

The per protocol fixed effects analysis indicated results consistent with the ITT analysis, with no significant overall difference in HD:CAS scores between the treatment groups at baseline ($\beta 2=-1.3373$, p=0.131), and a significant interaction effect in the quadratic (p=0.016) and cubic contrasts (p=0.006) (Supplemental figure 2). Similarly to ITT analysis, no difference between treatment groups was found at the end of treatment (p=0.179).

**Supplemental figure 2. Estimated marginal means of HD:CAS scores over time by treatment group in the PP analysis**

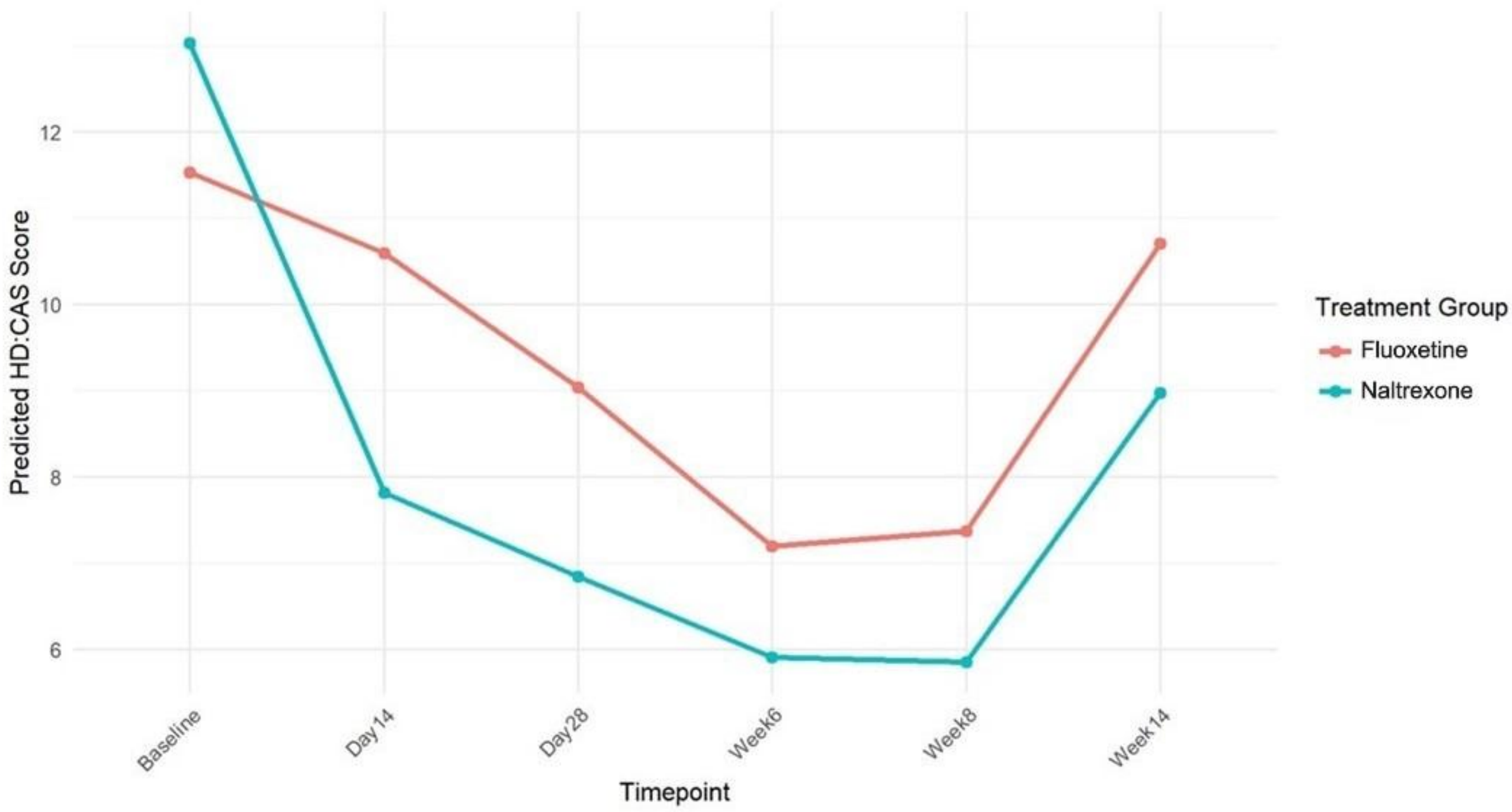


Post hoc pairwise comparisons in the fluoxetine group revealed that while there was a decrease in HD:CAS scores, none of the differences between time points were statistically significant (Supplemental table 5). For naltrexone, significant (p=<0.0001) reductions were observed at all time points compared to baseline, with the largest reduction by week 8 (7.19 points) (Supplemental table 6). Additionally, the analyses revealed a significant estimated increase of 3.13 points between week 8 and week 14.

**Supplemental table 5. Estimated change in HD:CAS score in the fluoxetine group over time: PP analysis**

| Contrast | Estimated change PP | p-value PP |
|---|---|---|
| Baseline to Day 14 | 0.9375 | 0.9943 |
| Baseline to Day 28 | 2.4926 | 0.7002 |
| Baseline to Week 6 | 4.3382 | 0.1234 |
| Baseline to Week 8 | 4.1691 | 0.1542 |
| Baseline to Week 14 | 0.8309 | 0.9968 |
| Day 14 to Day 28 | 1.5551 | 0.9461 |

| | | |
|---|---|---|
| Day 14 to Week 6 | 3.4007 | 0.3621 |
| Day 14 to Week 8 | 3.2316 | 0.4215 |
| Day 14 to Week 14 | -0.1066 | 1.0000 |
| Day 28 to Week 6 | 1.8456 | 0.8934 |
| Day 28 to Week 8 | 1.6765 | 0.9268 |
| Day 28 to Week 14 | -1.6618 | 0.9294 |
| Week 6 to Week 8 | -0.1691 | 1.0000 |
| Week 6 to Week 14 | -3.5074 | 0.3268 |
| Week 8 to Week 14 | -3.3382 | 0.3836 |

**Supplemental table 6. Estimated change in HD:CAS score in the naltrexone group over time: PP analysis**

| **Contrast** | **Estimated change PP** | **p-value PP** |
|---|---|---|
| Baseline to Day 14 | 5.2188 | **<0.0001** |
| Baseline to Day 28 | 6.1875 | **<0.0001** |
| Baseline to Week 6 | 7.1250 | **<0.0001** |
| Baseline to Week 8 | 7.1875 | **<0.0001** |
| Baseline to Week 14 | 4.0625 | **<0.0001** |
| Day 14 to Day 28 | 0.9688 | 0.8131 |
| Day 14 to Week 6 | 1.9062 | 0.1412 |
| Day 14 to Week 8 | 1.9688 | 0.1172 |
| Day 14 to Week 14 | -1.1562 | 0.6718 |
| Day 28 to Week 6 | 0.9375 | 0.8333 |
| Day 28 to Week 8 | 1.0000 | 0.7918 |
| Day 28 to Week 14 | -2.1250 | 0.0711 |
| Week 6 to Week 8 | 0.0625 | 1.0000 |
| Week 6 to Week 14 | -3.0625 | **0.0014** |

| | | |
|---|---|---|
| Week 8 to Week 14 | -3.1250 | **0.0010** |

**Supplemental table 7. Changes in raw mean values of HD:CAS over time by treatment group, ITT dataset**

| Treatment group | Time point | Mean | SD | N |
|---|---|---|---|---|
| Fluoxetine | Baseline | 13.10 | 3.94 | 40 |
| Fluoxetine | Day 14 | 7.32 | 5.03 | 40 |
| Fluoxetine | Day 28 | 6.15 | 4.99 | 40 |
| Fluoxetine | Week 6 | 5.22 | 5.26 | 40 |
| Fluoxetine | Week 8 | 4.68 | 4.94 | 40 |
| Fluoxetine | Week 14 | 7.35 | 5.84 | 40 |
| Naltrexone | Baseline | 14.50 | 2.96 | 40 |
| Naltrexone | Day 14 | 5.30 | 4.86 | 40 |
| Naltrexone | Day 28 | 4.82 | 4.51 | 40 |
| Naltrexone | Week 6 | 4.12 | 3.73 | 40 |
| Naltrexone | Week 8 | 4.72 | 4.93 | 40 |
| Naltrexone | Week 14 | 6.85 | 5.99 | 40 |

**Supplemental table 8. Changes in raw mean values of HD:CAS over time by treatment group, PP dataset**

| Treatment group | Time point | Mean | SD | N |
|---|---|---|---|---|
| Fluoxetine | Baseline | 13.00 | 3.75 | 32 |
| Fluoxetine | Day 14 | 7.81 | 4.97 | 32 |
| Fluoxetine | Day 28 | 6.84 | 5.02 | 32 |
| Fluoxetine | Week 6 | 5.91 | 5.26 | 32 |
| Fluoxetine | Week 8 | 5.84 | 4.87 | 32 |
| Fluoxetine | Week 14 | 8.97 | 5.29 | 32 |
| Naltrexone | Baseline | 14.50 | 3.00 | 34 |
| Naltrexone | Day 14 | 5.03 | 4.20 | 34 |
| Naltrexone | Day 28 | 4.65 | 3.81 | 34 |
| Naltrexone | Week 6 | 4.62 | 3.75 | 34 |
| Naltrexone | Week 8 | 4.32 | 4.12 | 34 |
| Naltrexone | Week 14 | 7.24 | 5.77 | 34 |

**HBI and SCS**

As the primary scale HD:CAS is not validated, two additional measures of CSBD were used. The Hypersexual Behaviour Inventory (HBI-19)[10] is a validated 19-item scale assessing hypersexual disorder based on the proposed criteria for DSM-5. Each item is rated on a 5-point Likert scale (1-5), resulting in a total score of 19 to 95, with a threshold of ≥53 points suggested as an indicator of CSBD. Sexual Compulsivity Scale (SCS)[11] measures sexual compulsivity with ten items, on a 4-point scale (1-4), with a total score of 10 to 40.

The correlation using Spearman's rho between HD:CAS and HBI at baseline was *r*=0.64, *p*<.001 and *r*=0.4, *p*<0.001 between HD:CAS and SCS. There was no significant overall difference in HBI scores between treatment groups at baseline ($\beta$2=-3.916, *p*=0.212) in the ITT analysis. There was a significant linear decrease in HBI across time points (*p*=0.0004). Marginally significant interactions suggest possible treatment group differences in the trajectory (linear *p*=0.060 and quadratic *p*=0.060) of HBI over time (Supplemental figure 3). No significant differences were found between the treatment groups at week 8 (*p*=0.301).

**Supplemental figure 3. HBI interaction effects ITT**

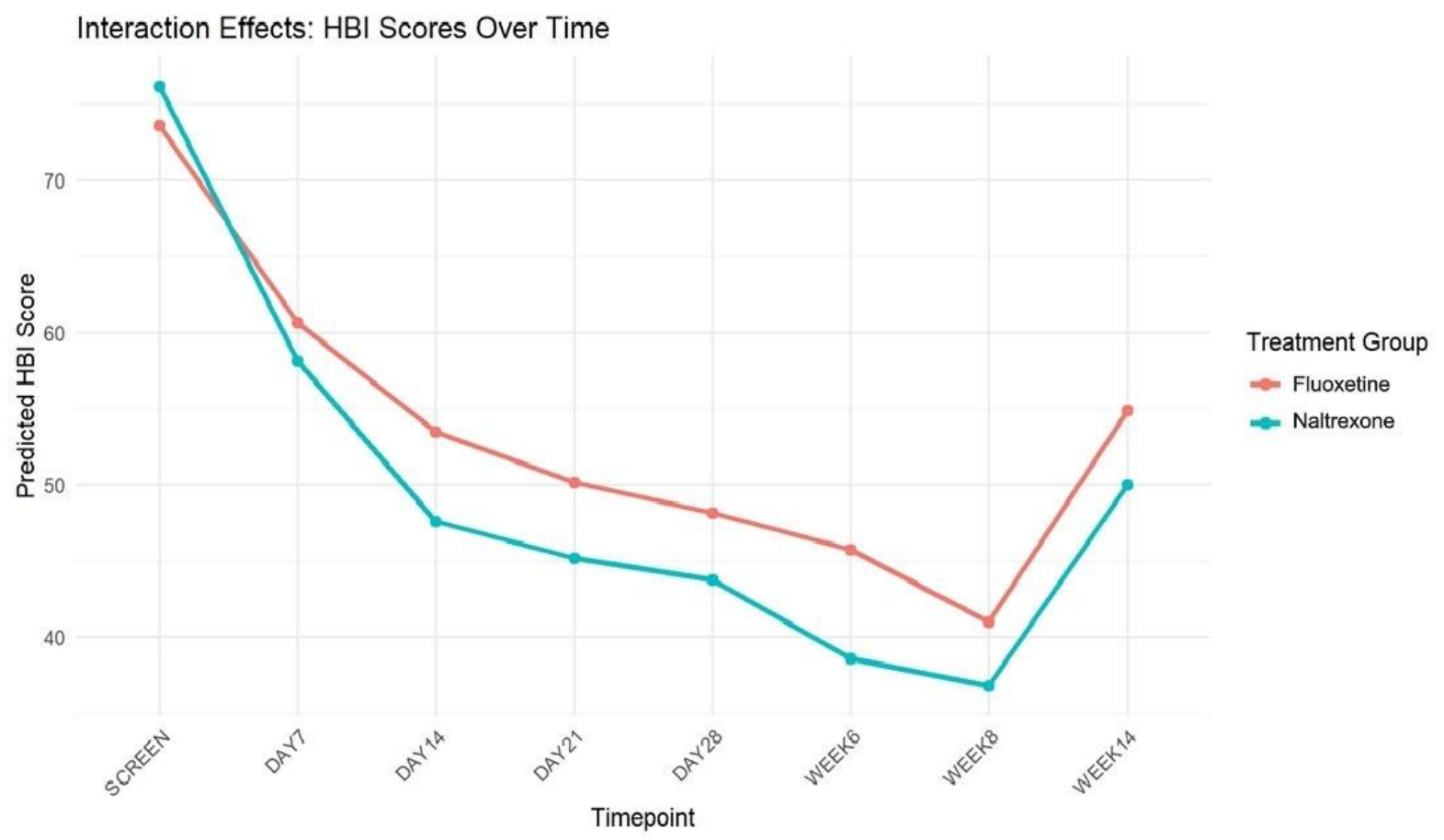


The PP analyses of HBI revealed no overall difference in HBI scores between treatment groups at baseline ($\beta$2=-4.669, *p*=0.167). However, there was a significant difference in the trajectory over time (linear *p*=0.019 and quadratic *p*=0.009) (Supplemental figure 4).

**Supplemental figure 4. HBI interaction effects PP**

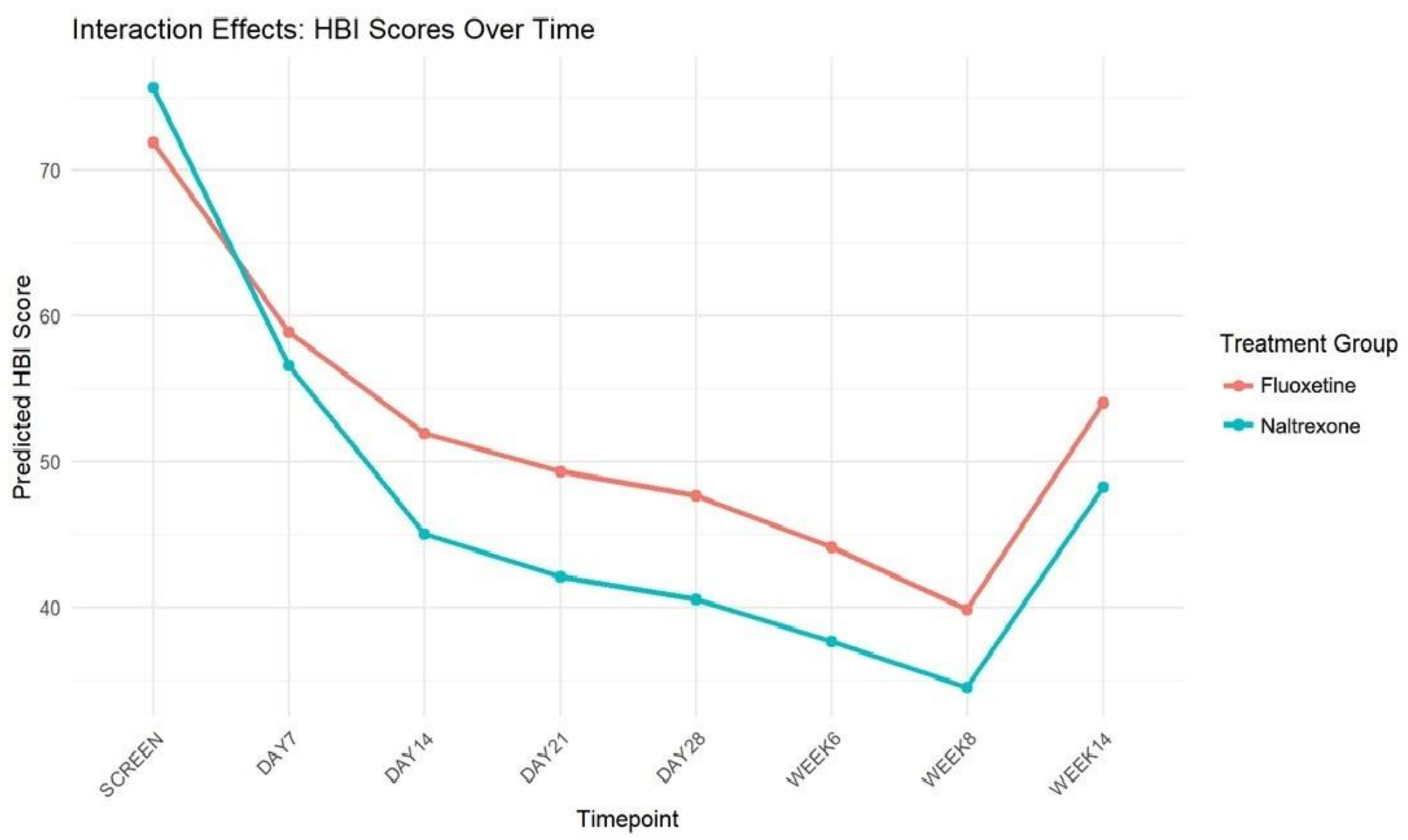

No significant differences in HBI scores were found between the treatment groups at the end of treatment (*p*=0.205). Significant reductions were observed from baseline across all subsequent time points (all p<0.0001), indicating a consistent improvement in HBI over time for both groups. The scores increased significantly after treatment discontinuation, suggesting a reduction in treatment benefit following cessation at week 8 (Supplemental Table 9 and 10).

**Supplemental table 9. Estimated change in HBI score over time in the fluoxetine group: ITT and PP analysis**

| **Contrast** | **Estimated change ITT** | **p-value ITT** | **Estimated change PP** | **p-value PP** |
|---|---|---|---|---|
| Baseline to Day 7 | 12.96 | **<0.0001** | 13.00 | **<0.0001** |
| Baseline to Day 14 | 20.09 | **<0.0001** | 19.91 | **<0.0001** |
| Baseline to Day 21 | 23.43 | **<0.0001** | 22.53 | **<0.0001** |
| Baseline to Day 28 | 25.43 | **<0.0001** | 24.19 | **<0.0001** |
| Baseline to Week 6 | 27.85 | **<0.0001** | 27.76 | **<0.0001** |
| Baseline to Week 8 | 32.59 | **<0.0001** | 32.01 | **<0.0001** |
| Baseline to Week 14 | 18.74 | **<0.0001** | 17.84 | **<0.0001** |
| Day 7 to Day 14 | 7.13 | 0.0853 | 6.91 | 0.1650 |
| Day 7 to Day 21 | 10.47 | **0.0014** | 9.53 | **0.0096** |
| Day 7 to Day 28 | 12.48 | **<0.0001** | 11.19 | **0.0010** |
| Day 7 to Week 6 | 14.89 | **<0.0001** | 14.76 | **<0.0001** |
| Day 7 to Week 8 | 19.63 | **<0.0001** | 19.01 | **<0.0001** |
| Day 7 to Week 14 | 5.78 | 0.3459 | 4.84 | 0.6127 |
| Day 14 to Day 21 | 3.34 | 0.8979 | 2.62 | 0.9769 |
| Day 14 to Day 28 | 5.35 | 0.4270 | 4.29 | 0.7572 |
| Day 14 to Week 6 | 7.76 | 0.0619 | 7.85 | 0.0731 |
| Day 14 to Week 8 | 12.50 | **0.0001** | 12.11 | **0.0002** |
| Day 14 to Week 14 | -1.35 | 0.9996 | -2.06 | 0.9945 |
| Day 21 to Day 28 | 2.00 | 0.9947 | 1.66 | 0.9987 |
| Day 21 to Week 6 | 4.42 | 0.7095 | 5.23 | 0.5261 |
| Day 21 to Week 8 | 9.16 | **0.0171** | 9.48 | **0.0114** |
| Day 21 to Week 14 | -4.69 | 0.6415 | -4.69 | 0.6523 |
| Day 28 to Week 6 | 2.41 | 0.9853 | 3.57 | 0.8950 |
| Day 28 to Week 8 | 7.15 | 0.1437 | 7.82 | 0.0807 |
| Day 28 to Week 14 | -6.69 | 0.1903 | -6.35 | 0.2677 |
| Week 6 to Week 8 | 4.74 | 0.6668 | 4.26 | 0.7721 |
| Week 6 to Week 14 | -9.11 | **0.0174** | -9.91 | **0.0065** |
| Week 8 to Week 14 | -13.85 | **<0.0001** | -14.17 | **<0.0001** |

**Supplemental table 10. Estimated change in HBI score in the naltrexone group over time: ITT and PP analysis**

| **Contrast** | **Estimated change ITT** | **p-value ITT** | **Estimated change PP** | **p-value PP** |
|---|---|---|---|---|
| Baseline to Day 7 | 18.01 | **<0.0001** | 19.06 | **<0.0001** |
| Baseline to Day 14 | 28.58 | **<0.0001** | 30.59 | **<0.0001** |
| Baseline to Day 21 | 30.94 | **<0.0001** | 33.53 | **<0.0001** |
| Baseline to Day 28 | 32.41 | **<0.0001** | 35.11 | **<0.0001** |
| Baseline to Week 6 | 37.55 | **<0.0001** | 38.00 | **<0.0001** |
| Baseline to Week 8 | 39.34 | **<0.0001** | 41.12 | **<0.0001** |
| Baseline to Week 14 | 26.17 | **<0.0001** | 27.37 | **<0.0001** |
| Day 7 to Day 14 | 10.57 | **0.0009** | 11.53 | **0.0003** |
| Day 7 to Day 21 | 12.93 | **<0.0001** | 14.47 | **<0.0001** |

| | | | | |
|---|---|---|---|---|
| Day 7 to Day 28 | 14.40 | **<0.0001** | 16.05 | **<0.0001** |
| Day 7 to Week 6 | 19.54 | **<0.0001** | 18.94 | **<0.0001** |
| Day 7 to Week 8 | 21.32 | **<0.0001** | 22.06 | **<0.0001** |
| Day 7 to Week 14 | 8.16 | **0.0346** | 8.31 | **0.0339** |
| Day 14 to Day 21 | 2.36 | 0.9836 | 2.94 | 0.9512 |
| Day 14 to Day 28 | 3.83 | 0.8070 | 4.52 | 0.6697 |
| Day 14 to Week 6 | 8.97 | **0.0114** | 7.41 | 0.0898 |
| Day 14 to Week 8 | 10.76 | **0.0007** | 10.53 | **0.0015** |
| Day 14 to Week 14 | -2.41 | 0.9826 | -3.22 | 0.9228 |
| Day 21 to Day 28 | 1.47 | 0.9992 | 1.58 | 0.9989 |
| Day 21 to Week 6 | 6.61 | 0.1710 | 4.47 | 0.6919 |
| Day 21 to Week 8 | 8.39 | **0.0241** | 7.59 | 0.0752 |
| Day 21 to Week 14 | -4.77 | 0.5953 | -6.16 | 0.2773 |
| Day 28 to Week 6 | 5.14 | 0.4866 | 2.89 | 0.9575 |
| Day 28 to Week 8 | 6.92 | 0.1223 | 6.01 | 0.2976 |
| Day 28 to Week 14 | -6.24 | 0.2418 | -7.74 | 0.0689 |
| Week 6 to Week 8 | 1.79 | 0.9970 | 3.12 | 0.9340 |
| Week 6 to Week 14 | -11.38 | **0.0004** | -10.63 | **0.0017** |
| Week 8 to Week 14 | -13.16 | **<0.0001** | -13.75 | **<0.0001** |

On SCS, the ITT and PP analysis showed no significant difference between the groups at baseline (for ITT: $\beta 2=-1.472$, $p=0.252$, for PP: $\beta 2=-1.412$, $p=0.308$). Both treatment groups significantly improved over time, however there was no significant treatment effect (for ITT: $p=0.427$, for PP: $p=0.377$) or interaction effect. A significant increase between week 8 and 14 was only observed in the PP analysis of fluoxetine (Supplemental figures 5 and 6, supplemental tables 11 and 12).

**Supplemental figure 5. SCS interaction effects ITT**

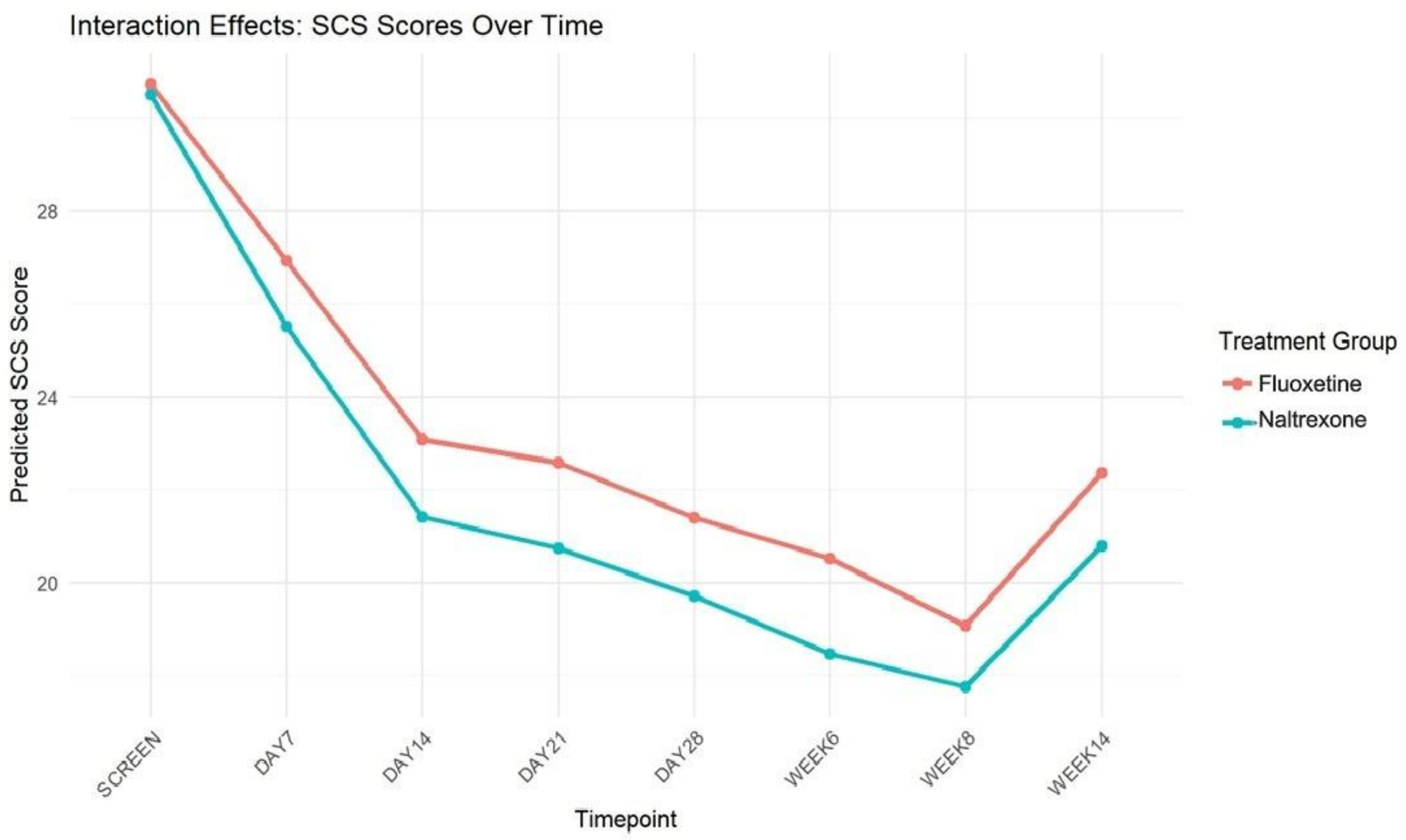

**Supplemental figure 6. SCS interaction effects PP**

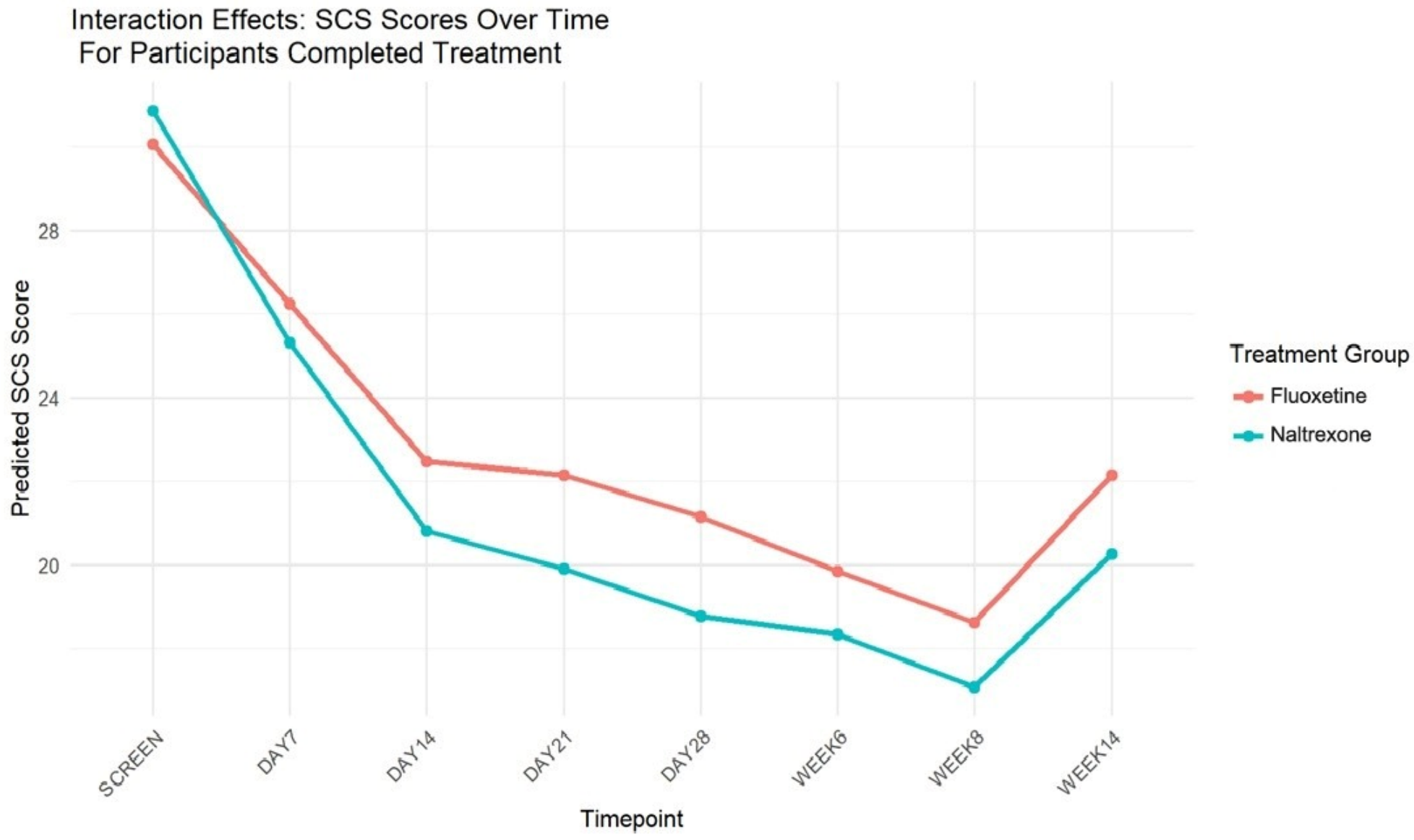


**Suplemental table 11. Estimated change in SCS score in the fluoxetine group over time: ITT and PP analysis**

| Contrast | Estimated change ITT | p-value ITT | Estimated change PP | p-value PP |
|---|---|---|---|---|
| Baseline to Day 7 | 3.7985 | **0.0060** | 3.812 | **0.0166** |
| Baseline to Day 14 | 7.6406 | **<0.0001** | 7.594 | **<0.0001** |
| Baseline to Day 21 | 8.1439 | **<0.0001** | 7.906 | **<0.0001** |
| Baseline to Day 28 | 9.3350 | **<0.0001** | 8.907 | **<0.0001** |
| Baseline to Week 6 | 10.2062 | **<0.0001** | 10.210 | **<0.0001** |
| Baseline to Week 8 | 11.6505 | **<0.0001** | 11.439 | **<0.0001** |
| Baseline to Week 14 | 8.3731 | **<0.0001** | 7.906 | **<0.0001** |
| Day 7 to Day 14 | 3.8421 | **0.0055** | 3.781 | **0.0182** |
| Day 7 to Day 21 | 4.3454 | **0.0013** | 4.094 | **0.0070** |
| Day 7 to Day 28 | 5.5364 | **<0.0001** | 5.095 | **0.0002** |
| Day 7 to Week 6 | 6.4076 | **<0.0001** | 6.398 | **<0.0001** |
| Day 7 to Week 8 | 7.8520 | **<0.0001** | 7.627 | **<0.0001** |
| Day 7 to Week 14 | 4.5745 | **0.0007** | 4.094 | **0.0070** |
| Day 14 to Day 21 | 0.5033 | 0.9998 | 0.312 | 1.0000 |
| Day 14 to Day 28 | 1.6943 | 0.7523 | 1.314 | 0.9423 |
| Day 14 to Week 6 | 2.5655 | 0.2566 | 2.616 | 0.2886 |
| Day 14 to Week 8 | 4.0099 | **0.0072** | 3.846 | **0.0167** |
| Day 14 to Week 14 | 0.7324 | 0.9975 | 0.312 | 1.0000 |
| Day 21 to Day 28 | 1.1910 | 0.9562 | 1.001 | 0.9873 |
| Day 21 to Week 6 | 2.0622 | 0.5649 | 2.304 | 0.4581 |
| Day 21 to Week 8 | 3.5066 | **0.0378** | 3.533 | **0.0398** |
| Day 21 to Week 14 | 0.2291 | 1.0000 | 0.000 | 1.0000 |
| Day 28 to Week 6 | 0.8712 | 0.9935 | 1.303 | 0.9472 |

| | | | | |
|---|---|---|---|---|
| Day 28 to Week 8 | 2.3156 | 0.4375 | 2.532 | 0.3424 |
| Day 28 to Week 14 | -0.9619 | 0.9882 | -1.001 | 0.9873 |
| Week 6 to Week 8 | 1.4444 | 0.9077 | 1.229 | 0.9613 |
| Week 6 to Week 14 | -1.8331 | 0.7208 | -2.304 | 0.4581 |
| Week 8 to Week 14 | -3.2775 | 0.0748 | -3.533 | **0.0398** |

**Supplemental table 12. Estimated change in SCS score in the naltrexone group over time: ITT and PP analysis**

| Contrast | Estimated change ITT | p-value ITT | Estimated change PP | p-value PP |
|---|---|---|---|---|
| Baseline to Day 7 | 4.9849 | **0.0001** | 5.559 | **<0.0001** |
| Baseline to Day 14 | 9.0930 | **<0.0001** | 10.059 | **<0.0001** |
| Baseline to Day 21 | 9.7617 | **<0.0001** | 10.980 | **<0.0001** |
| Baseline to Day 28 | 10.7895 | **<0.0001** | 12.102 | **<0.0001** |
| Baseline to Week 6 | 12.0473 | **<0.0001** | 12.533 | **<0.0001** |
| Baseline to Week 8 | 12.7416 | **<0.0001** | 13.794 | **<0.0001** |
| Baseline to Week 14 | 9.7071 | **<0.0001** | 10.605 | **<0.0001** |
| Day 7 to Day 14 | 4.1081 | **0.0026** | 4.500 | **0.0011** |
| Day 7 to Day 21 | 4.7768 | **0.0002** | 5.422 | **<0.0001** |
| Day 7 to Day 28 | 5.8046 | **<0.0001** | 6.543 | **<0.0001** |
| Day 7 to Week 6 | 7.0624 | **<0.0001** | 6.974 | **<0.0001** |
| Day 7 to Week 8 | 7.7568 | **<0.0001** | 8.235 | **<0.0001** |
| Day 7 to Week 14 | 4.7222 | **0.0003** | 5.046 | **0.0001** |
| Day 14 to Day 21 | 0.6687 | 0.9984 | 0.922 | 0.9907 |
| Day 14 to Day 28 | 1.6965 | 0.7469 | 2.043 | 0.5776 |
| Day 14 to Week 6 | 2.9543 | 0.1041 | 2.474 | 0.3208 |
| Day 14 to Week 8 | 3.6486 | **0.0127** | 3.735 | **0.0149** |
| Day 14 to Week 14 | 0.6141 | 0.9991 | 0.546 | 0.9997 |
| Day 21 to Day 28 | 1.0278 | 0.9788 | 1.121 | 0.9720 |
| Day 21 to Week 6 | 2.2855 | 0.3976 | 1.552 | 0.8555 |
| Day 21 to Week 8 | 2.9799 | 0.0924 | 2.814 | 0.1716 |
| Day 21 to Week 14 | -0.0546 | 1.0000 | -0.375 | 1.0000 |
| Day 28 to Week 6 | 1.2578 | 0.9396 | 0.431 | 0.9999 |
| Day 28 to Week 8 | 1.9521 | 0.5879 | 1.693 | 0.7835 |
| Day 28 to Week 14 | -1.0824 | 0.9731 | -1.496 | 0.8778 |
| Week 6 to Week 8 | 0.6944 | 0.9981 | 1.261 | 0.9451 |
| Week 6 to Week 14 | -2.3402 | 0.3762 | -1.928 | 0.6590 |
| Week 8 to Week 14 | -3.0346 | 0.0857 | -3.189 | 0.0735 |

The supplementary questionnaires HBI and SCS confirmed the results of the ITT analysis of HD:CAS, showing significant improvement over time in both treatment groups but no difference in the pattern of change. HD:CAS and HBI share the same theoretical framework but HD:CAS includes a specified timespan with items quantifying problematic sexual behaviours (eg, time spent, number of orgasms) which might be more sensitive to early changes. Although not consistently significant, a post-treatment symptom increase was observed in both groups, regardless of measure used, suggesting a potential loss of treatment effect after discontinuation.

**Supplemental table 13. Summary of adverse events (AE) (occurring in ≥5% of patients) by MedDRA system**

| | Fluoxetine (n = 39) | Naltrexone (n = 40) |
|---|---|---|
| Number of AE per person, median (range) | 3 (0-11) | 4 (0-18) |
| Patients with at least one grade 1-2 AE, No. (%) | 37 (95) | 37 (93) |
| Grade 3 AE, No. (%) | 2 (5) | 0 (0) |
| Nervous system disorders, No. (%) | | |
| Dizziness | 4 (10) | 14 (35) |
| Tremor | 4 (10) | 5 (13) |
| Headache | 8 (21) | 10 (25) |
| Disturbance in attention | 2 (5) | 1 (3) |
| General disorders and administration site conditions, No. (%) | | |
| Fatigue | 10 (26) | 23 (58) |
| Thirst | 0 (0) | 3 (8) |
| Investigations, No. (%) | | |
| Weight increased | 3 (8) | 2 (5) |
| Weight decreased | 6 (15) | 6 (15) |
| Urine output increased | 3 (8) | 1 (3) |
| Aspartate aminotransferase increased | 4 (10) | 1 (3) |
| Blood alkaline phosphatase increased | 1 (3) | 2 (5) |
| Gastrointestinal disorders, No. (%) | | |
| Dry mouth | 3 (8) | 3 (8) |
| Nausea | 6 (15) | 16 (40) |
| Abdominal pain | 0 (0) | 6 (15) |
| Dyspepsia | 3 (8) | 1 (3) |
| Flatulence | 2 (5) | 1 (3) |
| Diarrhoea | 7 (18) | 5 (13) |
| Constipation | 1 (3) | 3 (8) |
| Psychiatric disorders, No. (%) | | |
| Terminal insomnia | 5 (13) | 6 (15) |
| Anxiety | 4 (10) | 3 (8) |
| Depressed mood | 0 (0) | 2 (5) |
| Apathy | 3 (8) | 4 (10) |
| Abnormal dreams | 3 (8) | 6 (15) |
| Libido decreased | 28 (72) | 24 (60) |
| Reproductive system and breast disorders, No. (%) | | |
| Erectile dysfunction | 8 (21) | 6 (15) |
| Ejaculation delayed | 18 (46) | 7 (18) |
| Musculoskeletal and connective tissue disorders, No. (%) | | |
| Myalgia | 3 (8) | 3 (8) |
| Skin and subcutaneous tissue disorders, No. (%) | | |
| Rash | 2 (5) | 0 (0) |
| Pruritus | 3 (8) | 1 (3) |
| Hyperhidrosis | 2 (5) | 3 (8) |
| Cardiac disorders, No. (%) | | |
| Palpitations | 0 (0) | 2 (5) |
| All adverse events were noted in a paper log (at least one episode/event) with an assessment of severity (mild/moderate/severe), evaluation of causality related to treatment (likely/possibly/unlikely), and any actions taken in response. AEs presented in this table are assessed to be possibly or probably related to the drug. | | |

**Supplemental table 14. All treatment-emerged adverse events reported by patients (N=79), by MedDRA system organ class and preferred term**

| | Fluoxetine (n=39) | Naltrexone (n=40) |
|---|---|---|
| Nervous system disorders | | |
| Dizziness | 4 (10.3%) | 14 (35%) |
| Tremor | 4 (10.3%) | 5 (12.5%) |
| Headache | 8 (20.5%) | 10 (25%) |
| Disturbance in attention | 2 (5.1%) | 1 (2.5%) |
| Hypoaesthesia | 1 (2.6%) | 0 (0%) |
| Paraesthesia | 1 (2.6%) | 1 (2.5%) |
| Memory impairment | 1 (2.6%) | 0 (0%) |
| General disorders and administration site conditions | 10 (25.6%) | 23 (57.5%) |
| Fatigue | 0 (0%) | 3 (7.5%) |
| Thirst | | |
| Investigations | | |
| Weight increased | 3 (7.7%) | 2 (5%) |
| Weight decreased | 6 (15.4%) | 6 (15%) |
| Urine output increased | 3 (7.7%) | 1 (2.5%) |
| Aspartate aminotransferase increased | 4 (10.3%) | 1 (2.5%) |
| Blood alkaline phosphatase increased | 1 (2.6%) | 2 (5%) |
| Alanine aminotransferase increased | 1 (2.6%) | 0 (0%) |
| Blood sodium decreased | 1 (2.6%) | 0 (0%) |
| Gastrointestinal disorders | | |
| Dry mouth | 3 (7.7%) | 3 (7.5%) |
| Nausea | 6 (15.4%) | 16 (40%) |
| Abdominal pain | 0 (0%) | 6 (15%) |
| Dyspepsia | 3 (7.7%) | 1 (2.5%) |
| Flatulence | 2 (5.1%) | 1 (2.5%) |
| Diarrhoea | 7 (17.9%) | 5 (12.5%) |
| Constipation | 1 (2.6%) | 3 (7.5%) |
| Salivary hypersecretion | 0 (0%) | 1 (2.5%) |
| Psychiatric disorders | | |
| Terminal insomnia | 5 (12.8%) | 6 (15%) |
| Anxiety | 4 (10.3%) | 3 (7.5%) |
| Depressed mood | 0 (0%) | 2 (5%) |
| Apathy | 3 (7.7%) | 4 (10%) |
| Abnormal dreams | 3 (7.7%) | 6 (15%) |
| Libido decreased | 28 (71.8%) | 24 (60%) |
| Mood swings | 0 (0%) | 1 (2.5%) |
| Nervousness | 0 (0%) | 1 (2.5%) |
| Libido increased | 0 (0%) | 1 (2.5%) |
| Orgasmic sensation decreased | 1 (2.6%) | 1 (2.5%) |
| Reproductive system and breast disorders | | |
| Erectile dysfunction | 8 (20.5%) | 6 (15%) |
| Ejaculation delayed | 18 (46.2%) | 7 (17.5%) |
| Spontaneous penile erection | 0 (0%) | 1 (2.5%) |
| Spontaneous ejaculation | 1 (2.6%) | 1 (2.5%) |
| Musculoskeletal and connective tissue disorders | 3 (7.7%) | 3 (7.5%) |
| Myalgia | 1 (2.6%) | 0 (0%) |
| Arthralgia | 0 (0%) | 1 (2.5%) |
| Muscular weakness | 0 (0%) | 1 (2.5%) |
| Groin pain | | |
| Skin and subcutaneous tissue disorders | | |
| Rash | 2 (5.1%) | 0 (0%) |
| Pruritus | 3 (7.7%) | 1 (2.5%) |
| Hyperhidrosis | 2 (5.1%) | 3 (7.5%) |
| Urticaria | 1 (2.6%) | 0 (0%) |

| | | |
|---|---|---|
| Cardiac disorders<br>Palpitations | 0 (0%) | 2 (5%) |
| Eye Disorders<br>Vision blurred<br>Photophobia | 1 (2.6%)<br>1 (2.6%) | 1 (2.5%)<br>1 (2.5%) |
| Renal and urinary disorders<br>Urine flow decreased | 0 (0%) | 1 (2.5%) |
| Data are n (%). | | |

## eReferences

1. Savard J, Görts Öberg K, Dhejne C, et al. A randomized controlled trial of fluoxetine versus naltrexone in compulsive sexual behaviour disorder: presentation of the study protocol. *BMJ Open.* 2022;12:e051756. doi:10.1136/bmjopen-2021-051756

2. Adebahr R, Söderström EZ, Arver S, et al. Reaching men and women at risk of committing sexual offences: findings from the national Swedish telephone helpline PrevenTell. *J Sex Med.* 2021;18:1571-1581. doi:10.1016/j.jsxm.2021.06.007

3. Sheehan DV, Lecrubier Y, Sheehan KH, et al. The Mini-International Neuropsychiatric Interview (M.I.N.I.): the development and validation of a structured diagnostic psychiatric interview for DSM-IV and ICD-10. *J Clin Psychiatry.* 1998;59(suppl 20):22-33.

4. Posner K, Brown GK, Stanley B, et al. The Columbia–Suicide Severity Rating Scale: initial validity and internal consistency findings from three multisite studies with adolescents and adults. *Am J Psychiatry.* 2011;168:1266-1277. doi:10.1176/appi.ajp.2011.10111704

5. Kafka MP. Proposed dimensional measures for hypersexual disorder: the Hypersexual Disorder Screening Inventory (HDSI) and the Hypersexual Disorder Current Assessment Scale (HD:CAS). Unpublished manuscript. July 15, 2009. Revised December 2009.

6. Kafka MP. Hypersexual disorder: a proposed diagnosis for DSM-V. *Arch Sex Behav.* 2010;39:377-400. doi:10.1007/s10508-009-9574-7

7. Savard J, Öberg KG, Chatzittofis A, et al. Naltrexone in compulsive sexual behavior disorder: a feasibility study of twenty men. *J Sex Med.* 2020;17:1544-1552. doi:10.1016/j.jsxm.2020.04.316

8. Hallberg J, Kaldo V, Arver S, et al. Internet-administered cognitive behavioral therapy for hypersexual disorder, with or without paraphilia(s) or paraphilic disorder(s) in men: a pilot study. *J Sex Med.* 2020;17:2039-2054. doi:10.1016/j.jsxm.2020.06.013

9. Hallberg J, Kaldo V, Arver S, et al. A randomized controlled study of group-administered cognitive behavioral therapy for hypersexual disorder in men. *J Sex Med.* 2019;16:733-745. doi:10.1016/j.jsxm.2019.03.005

10. Reid RC, Garos S, Carpenter BN. Reliability, validity, and psychometric development of the Hypersexual Behavior Inventory in an outpatient sample of men. *Sex Addict Compulsivity.* 2011;18:30-51. doi:10.1080/10720162.2011.555709

11. Kalichman SC, Rompa D. Sexual sensation seeking and sexual compulsivity scales: reliability, validity, and predicting HIV risk behavior. *J Pers Assess.* 1995;65:586-601. doi:10.1207/s15327752jpa6503_16

12. Kinsey AC, Pomeroy WR, Martin CE. Sexual behavior in the human male. *Am J Public Health.* 2003;93:894-898. doi:10.2105/AJPH.93.6.894

13. Svanborg P, Åsberg M. A comparison between the Beck Depression Inventory and the self-rating version of the Montgomery Åsberg Depression Rating Scale. *J Affect Disord.* 2001;64:203-216. doi:10.1016/S0165-0327(00)00242-1

14. Kessler RC, Adler L, Ames M, et al. The World Health Organization adult ADHD self-report scale (ASRS): a short screening scale for use in the general population. *Psychol Med.* 2005;35:245-256. doi:10.1017/S0033291704002892

15. Saunders JB, Aasland OG, Babor TF, et al. Development of the Alcohol Use Disorders Identification Test (AUDIT): WHO collaborative project on early detection of persons with harmful alcohol consumption–II. *Addiction.* 1993;88:791-804. doi:10.1111/j.1360-0443.1993.tb02093.x

16. Bergman H, Källmén H. Alcohol use among Swedes and a psychometric evaluation of the Alcohol Use Disorders Identification Test. *Alcohol Alcohol.* 2002;37:245-251. doi:10.1093/alcalc/37.3.245

17. Berman AH, Bergman H, Palmstierna T, et al. Evaluation of the Drug Use Disorders Identification Test (DUDIT) in criminal justice and detoxification settings and in a Swedish population sample. *Eur Addict Res.* 2005;11:22-31. doi:10.1159/000081413

18. Molander O, Wennberg P, Berman AH. The Gambling Disorders Identification Test (GDIT): psychometric evaluation of a new comprehensive measure for gambling disorder and problem gambling. *Assessment.* 2023;30:225-237. doi:10.1177/10731911211045784

19. Lindström E, Lewander T, Malm U, et al. Patient-rated versus clinician-rated side effects of drug treatment in schizophrenia: clinical validation of a self-rating version of the UKU Side Effect Rating Scale (UKU-SERS-Pat). *Nord J Psychiatry.* 2001;55(suppl 44):5-69. doi:10.1080/080394801317085228
